# Methods for analyzing deep sequencing expression data: constructing the human and mouse promoterome with deepCAGE data

**DOI:** 10.1186/gb-2009-10-7-r79

**Published:** 2009-07-22

**Authors:** Piotr J Balwierz, Piero Carninci, Carsten O Daub, Jun Kawai, Yoshihide Hayashizaki, Werner Van Belle, Christian Beisel, Erik van Nimwegen

**Affiliations:** 1 Biozentrum, University of Basel, and Swiss Institute of Bioinformatics, Klingelbergstrasse 50/70, 4056-CH, Basel, Switzerland; 2 RIKEN Omics Science Center, RIKEN Yokohama Institute, 1-7-22 Suehiro-cho Tsurumi-ku Yokohama, Kanagawa, 230-0045 Japan; 3 Laboratory of Quantitative Genomics, Department of Biosystems Science and Engineering, Eidgenössische Technische Hochschule Zurich, Mattenstrasse 26, 4058 Basel, Switzerland

## Abstract

With the advent of ultra high-throughput sequencing technologies, increasingly researchers are turning to deep sequencing for gene expression studies. Here we present a set of rigorous methods for normalization, quantification of noise, and co-expression analysis of deep sequencing data. Using these methods on 122 cap analysis of gene expression (CAGE) samples of transcription start sites, we construct genome-wide 'promoteromes' in human and mouse consisting of a three-tiered hierarchy of transcription start sites, transcription start clusters, and transcription start regions.

## Abstract 2

A set of methods is presented for normalization, quantification of noise and co-expression analysis for gene expression studies using deep sequencing.

## Background

In recent years several technologies have become available that allow DNA sequencing at very high throughput - for example, 454 and Solexa. Although these technologies have originally been used for genomic sequencing, more recently researchers have turned to using these 'deep sequencing' or '(ultra-)high throughput' technologies for a number of other applications. For example, several researchers have used deep sequencing to map histone modifications genome-wide, or to map the locations at which transcription factors bind DNA (chromatin immunoprecipitation-sequencing (ChIP-seq)). Another application that is rapidly gaining attention is the use of deep sequencing for transcriptome analysis through the mapping of RNA fragments [1-4].

An alternative new high-throughput approach to gene expression analysis is cap analysis of gene expression (CAGE) sequencing [5]. CAGE is a relatively new technology introduced by Carninci and colleagues [6,7] in which the first 20 to 21 nucleotides at the 5' ends of capped mRNAs are extracted by a combination of cap trapping and cleavage by restriction enzyme *Mme*I. Recent development of the deepCAGE protocol employs the *Eco*P15 enzyme, resulting in approximately 27-nucleotide-long sequences. The 'CAGE tags' thus obtained can then be sequenced and mapped to the genome. In this way a genome-wide picture of transcription start sites (TSSs) at single base-pair resolution can be obtained. In the FANTOM3 project [8] this approach was taken to comprehensively map TSSs in the mouse genome. With the advent of deep sequencing technologies it has now become practical to sequence CAGE tag libraries to much greater depth, providing millions of tags from each biological sample. At such sequencing depths significantly expressed TSSs are typically sequenced a large number of times. It thus becomes possible to not only map the locations of TSSs but also quantify the expression level of each individual TSS [5].

There are several advantages that deep-sequencing approaches to gene expression analysis offer over standard micro-array approaches. First, large-scale full-length cDNA sequencing efforts have made it clear that most if not all genes are transcribed in different isoforms owing both to splice variation, alternative termination, and alternative TSSs [9]. One of the drawbacks of micro-array expression measurements has been that the expression measured by hybridization at individual probes is often a combination of expression of different transcript isoforms that may be associated with different promoters and may be regulated in different ways [10]. In contrast, because deep sequencing allows measurement of expression along the entire transcript the expression of individual transcript isoforms can, in principle, be inferred. CAGE-tag based expression measurements directly link the expression to individual TSSs, thereby providing a much better guidance for analysis of the regulation of transcription initiation. Other advantages of deep sequencing approaches are that they avoid the cross-hybridization problem that micro-arrays have [11], and that they provide a larger dynamic range.

However, whereas for micro-arrays there has been a large amount of work devoted to the analysis of the data, including issues of normalization, noise analysis, sequence-composition biases, background corrections, and so on, deep sequencing based expression analysis is still in its infancy and no standardized analysis protocols have been developed so far. Here we present new mathematical and computational procedures for the analysis of deep sequencing expression data. In particular, we have developed rigorous procedures for normalizing the data, a quantitative noise model, and a Bayesian procedure that uses this noise model to join sequence reads into clusters that follow a common expression profile across samples. The main application that we focus on in this paper is deepCAGE data. We apply our methodology to data from 66 mouse and 56 human CAGE-tag libraries. In particular, we identify TSSs genome-wide in mouse and human across a variety of tissues and conditions. In the first part of the results we present the new methods for analysis of deep sequencing expression data, and in the second part we present a statistical analysis of the human and mouse 'promoteromes' that we constructed.

## Results and Discussion

### Genome mapping

The first step in the analysis of deep-sequencing expression data is the mapping of the (short) reads to the genome from which they derive. This particular step of the analysis is not the topic of this paper and we only briefly discuss the mapping method that was used for the application to deepCAGE data. CAGE tags were mapped to the human (hg18 assembly) and mouse (mm8 assembly) genomes using a novel alignment algorithm called Kalign2 [12] that maps tags in multiple passes. In the first pass exactly mapping tags were recorded. Tags that did not match in the first pass were mapped allowing a single base substitution. In the third pass the remaining tags were mapped allowing indels. For the majority of tags there is a unique genome position to which the tag maps with least errors. However, if a tag matched multiple locations at a best match level, a multi-mapping CAGE tag rescue strategy developed by Faulkner *et al*. [13] was employed. For each tag that maps to multiple positions, a posterior probability is calculated for each of the possible mapping positions, which combines the likelihood of the observed error for each mapping with a prior probability for the mapped position. The prior probability for any position is proportional to the total number of tags that map to that position. As shown in [13], this mapping procedure leads to a significant increase in mapping accuracy compared to previous methods.

### Normalization

Once the RNA sequence reads or CAGE tags have been mapped to the genome we will have a (typically large) collection of positions for which at least one read/tag was observed. When we have multiple samples we will have, for each position, a read-count or tag-count profile that counts the number of reads/tags from each sample, mapping to that position. These tag-count profiles quantify the 'expression' of each position across samples and the simplest assumption would be that the true expression in each sample is simply proportional to the corresponding tag-count. Indeed, recent papers dealing with RNA-seq data simply count the number of reads/tags per kilobase per million mapped reads/tags [1]. That is, the tags are mapped to the annotated exonic sequences and their density is determined directly from the raw data. Similarly, previous efforts in quantifying expression from CAGE data [8] simply defined the 'tags per million' of a TSS as the number of CAGE tags observed at the TSS divided by the total number of mapped tags, multiplied by 1 million. However, such simple approaches assume that there are no systematic variations between samples (which are not controlled by the experimenter) that may cause the absolute tag-counts to vary across experiments. Systematic variations may result from the quality of the RNA, variation in library production, or even biases of the employed sequencing technology. To investigate this issue, we considered, for each sample, the distribution of tags per position.

**Figure 1:**
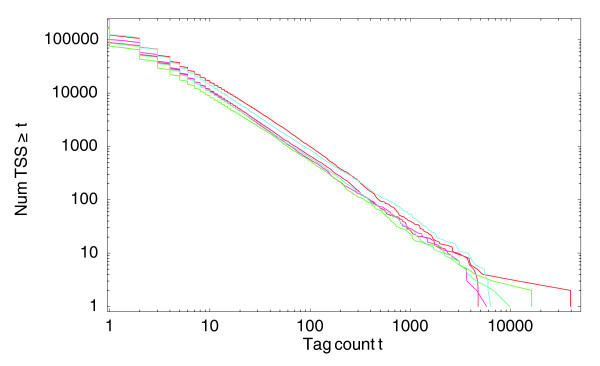
Reverse cumulative distributions for the number of different TSS positions that have at least a given number of tags mapping to them. Both axes are shown on a logarithmic scale. The three red curves correspond to the distributions of the three THP-1 cell control samples and the three blue curves to the three THP-1 samples after 24 hours of phorbol myristate acetate treatment. All other samples show very similar distributions (data not shown).

For our CAGE data the mapped tags correspond to TSS positions. Figure 1 shows reverse-cumulative distributions of the number of tags per TSS for six human CAGE samples that contain a total of a few million CAGE tags each. On the horizontal axis is the number of tag *t *and on the vertical axis the number of TSS positions to which at least *t *tags map. As the figure shows, the distributions of tags per TSS are power-laws to a very good approximation, spanning four orders of magnitude, and the slopes of the power-laws are a very similar across samples. These samples are all from THP-1 cells both untreated and after 24 hours of phorbol myristate acetate (PMA) treatment. Very similar distributions are observed for essentially all CAGE samples currently available (data not shown).

The large majority of observed TSSs have only a very small number of tags. These TSSs are often observed in only a single sample, and seem to correspond to very low expression 'background transcription'. On the other end of the scale there are TSSs that have as many as 10^4 ^tags, that is, close to 1% of all tags in the sample. Manual inspection confirms that these correspond to TSSs of genes that are likely to be highly expressed, for example, cytoskeletal or ribosomal proteins. It is quite remarkable in the opinion of these authors that both low expression background transcription, whose occurrence is presumably mostly stochastic, and the expression of the highest expressed TSSs, which is presumably highly regulated, occur at the extremes of a common underlying distribution. That this power-law expression distribution is not an artifact of the measurement technology is suggested by the fact that previous data from high-throughput serial analysis of gene expression (SAGE) studies have also found power-law distributions [14]. For ChIP-seq experiments, the number of tags observed per region also appears to follow an approximate power-law distribution [15]. In addition, our analysis of RNA-seq datasets from *Drosophila *shows that the number of reads per position follows an approximate power-law distribution as well (Figure S1 in Additional data file 1). These observations strongly suggest that RNA expression data generally obey power-law distributions. The normalization procedure that we present here should thus generally apply to deep sequencing expression data.

**Figure 2:**
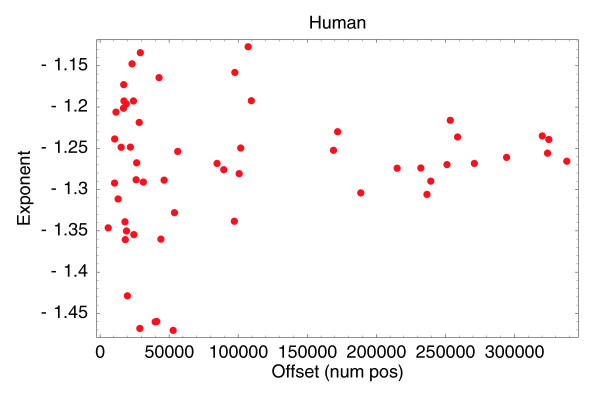
Fitted off-sets *n*_0 _(horizontal axis) and fitted exponents *α *(vertical axis) for the 56 human CAGE samples that have at least 100,000 tags.

For each sample, we fitted (see Materials and methods) the reverse-cumulative distribution of tags per TSS to a power-law of the form:

(1)

with *n*_0 _the inferred number of positions with at least *t *= 1 tag and *α *the slope of the power-law. Figure 2 shows the fitted values of *n*_0 _and *α *for all 56 human CAGE samples.

We see that, as expected, the inferred number of positions *n*_0 _varies significantly with the depth of sequencing; that is, the dots on the right are from the more recent samples that were sequenced in greater depth. In contrast, the fitted exponents vary relatively little around an average of approximately -1.25, especially for the samples with large numbers of tags.

**Figure 3:**
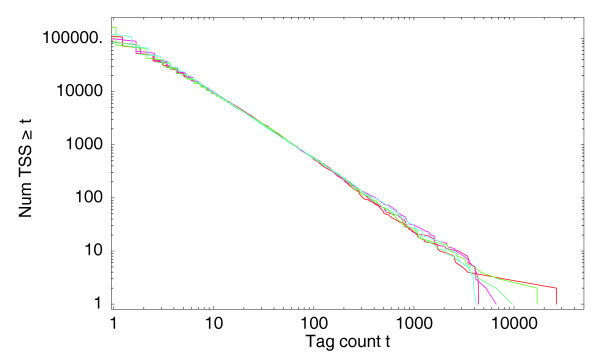
Normalized reverse cumulative distributions for the number of different TSS positions that have at least a given number of tags mapping to them. Both axes are shown on a logarithmic scale. The three red curves correspond to the distributions of the three THP-1 control samples and the three blue curves to the three THP-1 samples after 24 hours of PMA treatment.

In the analysis of micro-array data it has become accepted that it is beneficial to use so-called quantile normalization, in which the expression values from different samples are transformed to match a common reference distribution [16]. We follow a similar approach here. We make the assumption that the 'true' distribution of expression per TSS is really the same in all samples, and that the small differences in the observed reverse-cumulative distributions are the results of experimental biases that are varying across samples. This includes fluctuations in the fraction of tags that maps successfully, variations in sequence-specific linker efficiency, the noise in PCR amplification, and so on. To normalize our tag count, we map all tags to a reference distribution. We chose as reference distribution a power-law with an exponent of *α *= -1.25 and, for convenience, we chose the offset *n*_0 _such that the total number of tags is precisely 1 million. We then used the fits for all samples to transform the tag-counts into normalized 'tags per million' (TPM) counts (see Materials and methods). Figure 3 shows the same six distributions as in Figure 1, but now after the normalization.

Although the changes that this normalization introduces are generally modest, the collapse of the distributions shown in Figure 3 strongly suggests that the normalization improves quantitative comparability of the expression profiles. Indeed, as described below, for a replicate data-set in which two deepCAGE libraries were constructed from a common mRNA sample, the normalization significantly reduces the apparent variation between the replicates' expression profiles. Finally, we note that normalization to a common power-law distribution has also been proposed for normalizing micro-arrays [17].

In the remainder we will use the normalized tag counts to compare the expression at individual positions in the genome across samples. We also retain the raw tag-counts because, as we will see below, the noise on the observed tag count depends on these raw counts.

### Noise model

In order to analyze expression profiles, it is necessary to analyze the distribution of the noise on deepCAGE and other deep-sequencing expression measurements. To our knowledge, such an analysis has not yet been performed. Instead of determining noise on expression measurements, existing work has focused on defining models of the background distribution of tags/reads, which can be used to identify regions that have significantly more mapped tags/reads than expected from the background model. These background models assume that the number of tags in a given region follows either a simple Poisson distribution, or a Poisson distribution with gamma-distributed rate [18].

**Figure 4:**
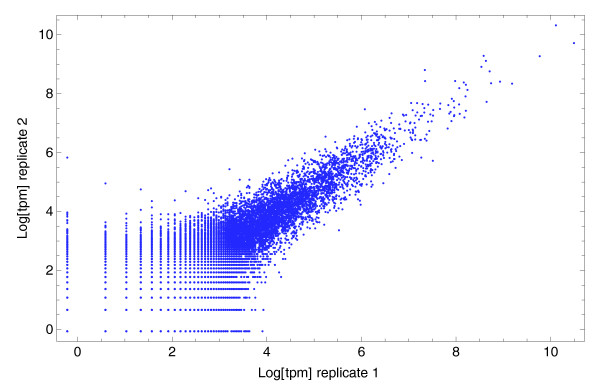
CAGE replicate from THP-1 cells after 8 hours of lipopolysaccharide treatment. For each position with mapped tags, the logarithm of the number of tags per million (TPM) in the first replicate is shown on the horizontal axis, and the logarithm of the number of TPM in the second replicate on the vertical axis. Logarithms are natural logarithms.

To quantitatively investigate the noise in the expression measurements, we compared tag-counts across replicate data-sets. Among the currently available CAGE data-sets there is one pair in which two libraries were prepared from a common mRNA sample and Figure 4 shows a scatter plot of the normalized tag counts (TPM) from the replicate measurements.

The figure shows that, at high TPM (that is, for positions with TPMs larger than *e*^4 ^≈ 55), the scatter has an approximately constant width whereas at low TPM the width of the scatter increases dramatically. This kind of funnel shape is familiar from micro-array expression data where the increase in noise at low expression is caused by the contribution of non-specific background hybridization. However, for the deepCAGE data this noise is of an entirely different origin.

In deep sequencing experiments the noise comes from essentially two separate processes. First, there is the noise that is introduced in going from the biological input sample to the final library that goes into the sequencer. Second, there is the noise introduced by the sequencing itself. For the CAGE experiments the former includes cap-trapping, linker ligation, cutting by the restriction enzyme, PCR amplification, and concatenation of the tags. In other deep-sequencing experiments, for example, RNA-seq or ChIP-seq with Solexa sequencing, there will similarly be processes such as the shearing or sonication of the DNA, adding of the linkers, and growing clusters on the surface of the flow cell.

With respect to the noise introduced by the sequencing itself, it seems reasonable to assume that the *N *tags that are eventually sequenced can be considered a random sample of size *N *of the material that went into the sequencer. This will lead to relatively large 'sampling' noise for tags that form only a small fraction of the pool. For example, assume that a particular tag has fraction *f *in the tag pool that went into the sequencer. This tag is expected to be sequenced ⟨*n*⟩ = *fN *times among the *N *sequenced tags, and the actual number of times *n *that it is sequenced will be Poisson distributed according to:

(2)

Indeed, recent work [19] shows that the noise in Solexa sequencing itself (that is, comparing different lanes of the same run) is Poisson distributed. It is clear, however, that the Poisson sampling is not the only source of noise. In Figure 4 there is an approximately fixed width of the scatter even at very high tag-counts, where the sampling noise would cause almost no difference in log-TPM between replicates. We thus conclude that, besides the Poisson sampling, there is an additional noise in the log-TPM whose size is approximately independent of the total log-TPM. Note that noise of a fixed size on the log-TPM corresponds to multiplicative noise on the level of the number of tags. It is most plausible that this multiplicative noise is introduced by the processes that take the original biological samples into the final samples that are sequenced; for example, linker ligation and PCR amplification may vary from tag to tag and from sample to sample. The simplest, least biased noise distribution, assuming only a fixed size of the noise, is a Gaussian distribution [20].

We thus model the noise as a convolution of multiplicative noise, specifically a Gaussian distribution of log-TPM with variance *σ*^2^, and Poisson sampling. As shown in the methods, if *f *is the original frequency of the TSS in the mRNA pool, and a total of *N *tags are sequenced, then the probability to obtain the TSS *n *times is approximately:

(3)

where the variance *σ*^2^(*n*) is given by:

(4)

That is, the measured log-TPM is a Gaussian whose mean matches the log-TPM in the input sample, with a variance equal to the variance of the multiplicative noise (*σ*^2^) plus one over the raw number of measured tags. The approximation (Equation 3) breaks down for *n *= 0. The probability to obtain *n *= 0 tags is approximately given by (Materials and methods):

(5)

We used the CAGE technical replicate (Figure 4) to estimate the variance *σ*^2 ^of the multiplicative noise (Materials and methods) and find *σ*^2 ^= 0.085. To illustrate the impact of the normalization, determining *σ*^2 ^on the same unnormalized data-set, we obtained *σ*^2 ^= 0.11, that is, a 29% increase in the apparent noise between the replicates. In addition to this replicate, among the human CAGE data-sets there is a time course of THP-1 cells after PMA treatment, measured in triplicate, which includes samples before PMA treatment and after only 1 hour of PMA treatment. Manual inspection shows that the correlation of tags per TSS for these two samples is as large as for the technical replicate. This makes sense because, on the time scale of 1 hour, the expression of most transcripts can probably not change appreciably [21]. Using a procedure (Materials and methods) that takes into account that a small fraction of TSSs may change expression significantly between the two samples, we estimated *σ*^2 ^as well for the three 0/1 hour sample pairs. The values we estimate are, respectively, *σ*^2 ^= 0.048, *σ*^2 ^= 0.116, and *σ*^2 ^= 0.058.

In summary, using four pairs of samples that are (almost) replicates, we find estimates of *σ*^2 ^ranging from 0.048 to 0.116. Although this analysis provides some evidence that the size of the multiplicative noise varies between samples, the range of inferred values is small and we will make the assumption that *σ*^2 ^is the same for all samples. As an estimate of *σ*^2 ^we took an intermediate value of *σ*^2 ^= 0.06 for the rest of our CAGE analysis.

**Figure 5:**
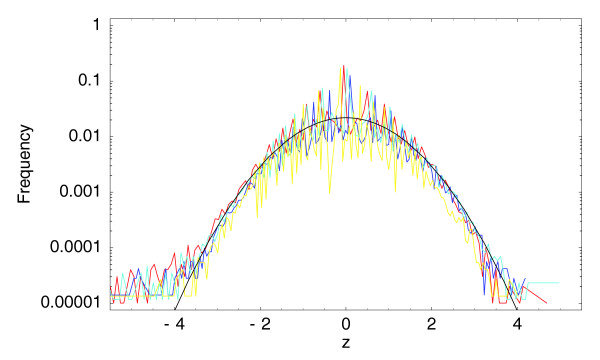
Observed histograms of *z*-statistics for the three 0/1 hour (in red, dark blue, and light blue) samples and for the technical replicate (in yellow) compared with the standard unit Gaussian (in black). The vertical axis is shown on a logarithmic scale.

We next validated this noise model as follows. According to our noise model, for TSSs that have non-zero expression in both samples, the z-statistic:

(6)

with *m' *the normalized expression at 1 hour and *n' *at zero hours, should be Gaussian distributed with standard deviation 1 (Materials and methods). We tested this for the three biological replicates at 0/1 hour and for the technical replicate. Figure 5 shows this theoretical distribution (in black) together with the observed histogram of *z*-values for the four replicates.

Although the data are noisy, it is clear that all three curves obey a roughly Gaussian distribution. Note the deviation from the theoretical curve at very low *z*, that is, *z *< -4, which appears only for the 0/1 hour comparisons. These correspond to the small fraction of positions that are significantly up-regulated at 1 hour. In summary, Figure 5 clearly shows that the data from the replicate experiments are well described by our noise model.

To verify the applicability of our noise model to RNA-seq data, we used two replicate data sets of *Drosophila *mRNA samples that were sequenced using Solexa sequencing and estimated a value of *σ*^2 ^= 0.073 for these replicate samples (Figure S2 in Additional data file 1). This fitted value of *σ*^2 ^is similar to those obtained for the CAGE samples.

Finally, the *σ*^2 ^values that we infer for the deep sequencing data are somewhat larger than what one typically finds for replicate expression profiles as measured by micro-arrays. However, it is important to stress that CAGE measures expression of individual TSSs, that is, single positions on the genome, whereas micro-arrays measure the expression of an entire gene, typically by combining measurements from multiple probes along the gene. Therefore, the size of the 'noise' in CAGE and micro-array expression measurements cannot be directly compared. For example, when CAGE measurements from multiple TSSs associated with the same gene are combined, expression profiles become significantly less noisy between replicates (*σ*^2 ^= 0.068 versus *σ*^2 ^= 0.085; Figures S4 and S5 in Additional data file 1). This applies also to RNA-seq data (*σ*^2 ^= 0.02 versus *σ*^2 ^= 0.073; Figure S2 and S3 in Additional data file 1).

### Promoterome construction

Using the methods outlined above on CAGE data, we can comprehensively identify TSSs genome-wide, normalize their expression, and quantitatively characterize the noise distribution in their expression measurements. This provides the most detailed information on transcription starts and, from the point of view of characterizing the transcriptome, there is, in principle, no reason to introduce additional analysis.

However, depending on the problem of interest, it may be useful to introduce additional filtering and/or clustering of the TSSs. For example, whereas traditionally it has been assumed that each 'gene' has a unique promoter and TSS, large-scale sequence analyses, such as performed in the FANTOM3 project [8], have made it clear that most genes are transcribed in different isoforms that use different TSSs. Alternative TSSs not only involve initiation from different areas in the gene locus - for example, from different starting exons - but TSSs typically come in local clusters spanning regions ranging from a few to over 100 bp wide.

These observations raise the question as to what an appropriate definition of a 'basal promoter' is. Should we think of each individual TSS as being driven by an individual 'promoter', even for TSSs only a few base-pairs apart on the genome? The answer to this question is a matter of definition and the appropriate choice depends on the application in question. For example, for the FANTOM3 study the main focus was to characterize all distinct regions containing a significant amount of transcription initiation. To this end the authors simply clustered CAGE tags whose genomic mappings overlapped by at least 1 bp [8]. Since CAGE tags are 20 to 21 bp long, this procedure corresponds to single-linkage clustering of TSSs within 20 to 21 bp of each other. A more recent publication [22] creates a hierarchical set of promoters by identifying all regions in which the density of CAGE tags is over a given cut-off. This procedure thus allows one to identify all distinct regions with a given total amount of expression for different expression levels and this is clearly an improvement over the *ad hoc *clustering method employed in the FANTOM3 analysis.

Both clustering methods just mentioned cluster CAGE tags based only on the overall density of mapped tags along the genome - that is, they ignore the expression profiles of the TSSs across the different samples. However, a key question that one often aims to address with transcriptome data is how gene expression is regulated. That is, whereas these methods can successfully identify the distinct regions from which transcription initiation is observed, they cannot detect whether the TSSs within a local cluster are similarly expressed across samples or that different TSSs in the cluster have different expression profiles. Manual inspection shows that, whereas there are often several nearby TSSs with essentially identical expression profiles across samples/tissues, one also finds cases in which TSSs that are only a few base-pairs apart show clearly distinct expression profiles. We hypothesize that, in the case of nearby co-expressed TSSs, the regulatory mechanisms recruit the RNA polymerase to the particular area on the DNA but that the final TSS that is used is determined by an essentially stochastic (thermodynamic) process. One could, for example, imagine that the polymerase locally slides back and forth on the DNA and chooses a TSS based on the affinity of the polymerase for the local sequence, such that different TSSs in the area are used in fixed relative proportions. In contrast, when nearby TSSs show different expression profiles one could imagine that there are particular regulatory sites that control initiation at individual TSSs.

Whatever the detailed regulatory mechanisms are, it is clear that, for the study of transcription regulation, it is important to properly separate local clusters of TSSs that are co-regulated from those that show distinct expression profiles. Below we present a Bayesian methodology that clusters nearby TSSs into 'transcription start clusters' (TSCs) that are co-expressed in the sense that their expression profiles are statistically indistinguishable.

A second issue is that, as shown by the power-law distribution of tags per TSS (Figure 1), we find a very large number of different TSSs used in each sample and the large majority of these have very low expression. Many TSSs have only one or a few tags and are often observed in one sample only. From the point of view of studying the regulation of transcription, it is clear that one cannot meaningfully speak of 'expression profiles' of TSSs that were observed only once or twice and only in one sample. That is, there appears to be a large amount of 'background transcription' and it is useful to separate these TSSs that are used very rarely, and presumably largely stochastically, from TSSs that are significantly expressed in at least one sample. Below we also provide a simple method for filtering such 'background transcription'.

Finally, for each significantly expressed TSC there will be a 'proximal promoter region' that contains regulatory sites that control the rate of transcription initiation from the TSSs within the TSC. Since TSCs can occur close to each other on the genome, individual regulatory sites may sometimes be controlling multiple nearby TSCs. Therefore, in addition to clustering nearby TSSs that are co-expressed, we introduce an additional clustering layer, in which TSCs with overlapping proximal promoters are clustered into 'transcription start regions' (TSRs). Thus, whereas different TSSs may share regulatory sites, the regulatory sites around a TSR only control the TSSs within the TSR.

Using the normalization method and noise model described above, we have constructed comprehensive 'promoteromes' of the human and mouse genomes from 122 CAGE samples across different human and mouse tissues and conditions (Materials and methods) by first clustering nearby co-regulated TSSs; second, filtering out background transcription; third, extracting proximal promoter regions around each TSS cluster; and fourth merging TSS clusters with overlapping proximal promoters into TSRs. We now describe each of these steps in the promoterome construction.

#### Clustering adjacent co-regulated transcription start sites

We define TSCs as sets of contiguous TSSs on the genome, such that each TSS is relatively close to the next TSS in the cluster, and the expression profiles of all TSSs in the cluster are indistinguishable up to measurement noise. To construct TSCs fitting this definition, we will use a Bayesian hierarchical clustering procedure that has the following ingredients. We start by letting each TSS form a separate, 1-bp wide TSC. For each pair of neighboring TSCs there is prior probability *π *(*d*) that these TSCs should be fused, which depends on the distance *d *along the genome between the two TSCs. For each pair of TSCs we calculate the likelihoods of two models for the expression profiles of the two TSCs. The first model assumes that the two TSCs have a constant relative expression in all samples (up to noise). The second model assumes that the two expression profiles are independent. Combining the prior *π *(*d*) and likelihoods of the two models, we calculate, for each contiguous pair of TSCs, a posterior probability that the two TSCs should be fused. We identify the pair with highest posterior probability and if this posterior probability is at least 1/2, we fuse this pair and continue clustering the remaining TSCs. Otherwise the clustering stops.

The details of the clustering procedure are described in Materials and methods. Here we will briefly outline the key ingredients. The key quantity for the clustering is the likelihood ratio of the expression profiles of two neighboring TSCs under the assumptions that their expression profiles are the same and independent, respectively. That is, if we denote by *x*_*s *_the logarithm of the TPM in sample *s *of one TSC, and by *y*_*s *_the log-TPM in sample *s *of a neighboring TSC, then we want to calculate the probability *P*({*x*_*s*_}, {*y*_*s*_}) of the two expression profiles assuming the two TSCs are expressed in the same way, and the probability *P*({*x*_*s*_}), *P*({*y*_*s*_}) of the two expression profiles assuming they are independent.

For a single TSS we write *x*_*s *_as the sum of a mean expression *μ*, the sample-dependent deviation *δ*_*s *_from this mean, and a noise term:

(7)

The probability *P*(*x*_*s*_|*μ *+ *δ*_*s*_) is given by the noise-distribution (Equation 3). To calculate the probability *P*({*x*_*s*_}) of the expression profile, we assume that the prior probability *P*(*μ*) of *μ *is uniformly distributed and that the prior probabilities of the *δ*_*s *_are drawn from a Gaussian with variance *α*, that is:

(8)

The probability of the expression profile of a single TSC is then given by integrating out the unknown 'nuisance' variables {*δ*_*s*_} and *μ*:

(9)

The parameter *α*, which quantifies the *a priori *expected amount of expression variance across samples, is determined by maximizing the joint likelihood of all TSS expression profiles (Materials and methods).

To calculate the probability *P*({*x*_*s*_}, {*y*_*s*_}), we assume that even though the two TSCs may have different mean expressions, their deviations *δ*_*s *_are the same across all samples. That is, we write:

(10)

and

(11)

The probability *P*({*x*_*s*_}, {*y*_*s*_}) is then given by integrating out the nuisance parameters:

(12)

As shown in the Materials and methods section, the integrals in Equations 9 and 12 can be done analytically. For each neighboring pair of TSCs we can thus analytically determine the log-ratio:

(13)

**Figure 6:**
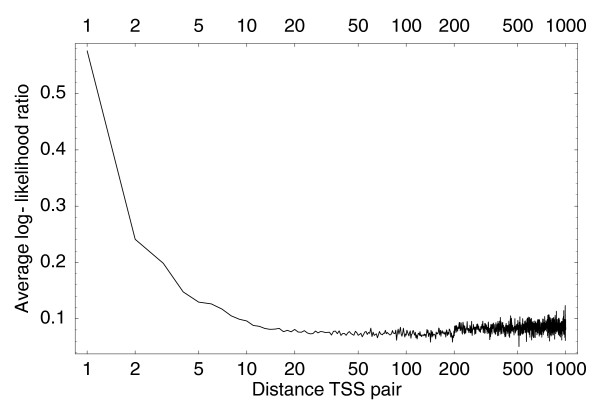
Average log-ratio *L *(Equation 13) for neighboring pairs of individual TSSs as a function of the distance between the TSSs. The horizontal axis is shown on a logarithmic scale.

To perform the clustering, we also need a prior probability that two neighboring TSCs should be fused and we will assume that this prior probability depends only on the distance between the two TSCs along the genome. That is, for closely spaced TSC pairs we assume it is *a priori *more likely that they are driven by a common promoter than for distant pairs of TSCs. To test this, we calculated the log-ratio *L *of Equation 13 for each consecutive pair of TSSs in the human CAGE data. Figure 6 shows the average of *L *as a function of the distance of the neighboring TSSs.

Figure 6 shows that the closer the TSSs, the more likely they are to be co-expressed. Once TSSs are more than 20 bp or so apart, they are not more likely to be co-expressed than TSSs that are very far apart. To reflect these observations, we will assume that the prior probability *π *(*d*) that two neighboring TSCs are co-expressed falls exponentially with their distance *d*, that is:

(14)

where *l *is a length-scale that we set to *l *= 10.

For each consecutive pair of TSCs we calculate *L *and we calculate a prior log-ratio:

(15)

where the distance *d *between two TSCs is defined as the distance between the most highly expressed TSSs in the two TSCs. We iteratively fuse the pair of TSCs for which *L *+ *R *is largest. After each fusion we of course need to update *R *and *L *for the neighbors of the fused pair. We keep fusing pairs until there is no longer any pair for which *L *+ *R *> 0 (corresponding to a posterior probability of 0.5 for the fusion).

#### Filtering background transcription

If one were principally interested in identifying all transcription initiation sites in the genome, one would of course not filter the set of TSCs obtained using the clustering procedure just described. However, when one is interested in studying regulation of expression then one would want to consider only those TSCs that show a substantial amount of expression in at least one sample and remove 'background transcription'. To this end we have to determine a cut-off on expression level to separate background from significantly expressed TSCs. As the distribution of expression per TSS does not naturally separate into a high expressed and low expressed part - that is, it is power-law distributed - this filtering is, to some extent, arbitrary.

According to current estimates, there are a few hundred thousand mRNAs per cell in mammals. In our analysis we have made the choice to retain all TSCs such that, in at least one sample, at least ten TPM derive from this TSC, that is, at least 1 in 100,000 transcripts. With this conservative cut-off we ensure that there is at least one mRNA per cell in at least one sample. Since for some samples the total number of tags is close to 100,000, a TSC may spuriously pass this threshold by having only 2 tags in a sample with low total tag count. To avoid these, we also demand that the TSC has one tag in at least two different samples.

#### Proximal promoter extraction and transcription start region construction

**Figure 7:**
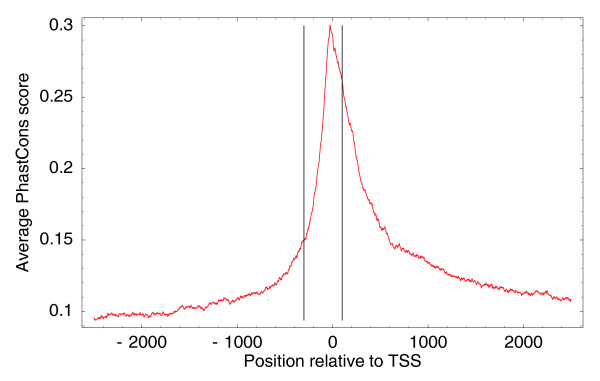
Average PhastCons (conservation) score relative to TSSs of genomic regions upstream and downstream of all human TSCs. The vertical lines show positions -300 and +100 with respect to TSSs.

Finally, for each of the TSCs we want to extract a proximal promoter region that contains regulatory sites that control the expression of the TSC, and, in addition, we want to cluster TSCs with overlapping proximal promoter regions. To estimate the typical size of the proximal promoters, we investigated conservation statistics in the immediate neighborhood of TSCs. For each human TSC we extracted PhastCons [23] scores 2.5 kb upstream and downstream of the highest expressed TSS in the TSC and calculated average PhastCons scores as a function of position relative to TSS (Figure 7).

We observe a sharp peak in conservation around the TSS, suggesting that the functional regulatory sites are highly concentrated immediately around it. Upstream of the TSS the conservation signal decays within a few hundred base-pairs, whereas downstream of the TSS the conservation first drops sharply and then more slowly. The longer tail of conservation downstream of the TSS is most likely due to selection on the transcript rather than on transcription regulatory sites.

Based on these conservation statistics, we conservatively chose the region from -300 to +100 with respect to the TSS as the proximal promoter region. Although the precise boundaries are, to some extent, arbitrary, it is clear that the conserved region peaks in a narrow region of only a few hundred base-pairs wide around the TSS. As a final step in the construction of the promoteromes, we clustered together all TSCs whose proximal promoter regions (that is, from 300 bp upstream of the first TSS in the TSC to 100 bp downstream of the last TSS in the TSC) overlap into TSRs.

### Promoterome statistics

To characterize the promoteromes that we obtained, we compared them with known annotations and we determined a number of key statistics.

#### Comparison with starts of known transcripts

**Figure 8:**
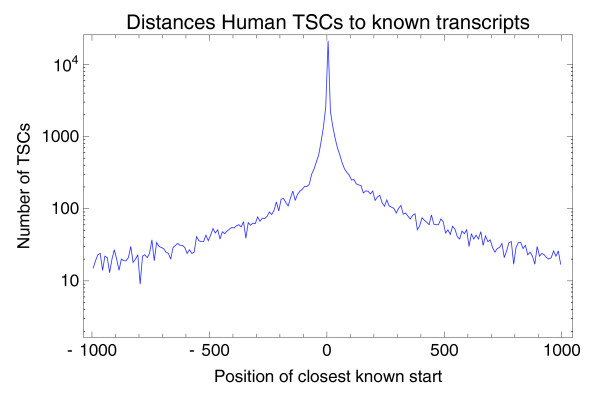
The number of TSCs as a function of their position relative to the nearest known mRNA start. Negative numbers mean the nearest known mRNA start is upstream of the TSC. The vertical axis is shown on a logarithmic scale. The figure shows only the 46,293 TSCs (62.3%) that have a known mRNA start within 1,000 bp.

Using the collection of all human mRNAs from the UCSC database [24], we compared the location of our TSCs with known mRNA starts. For each TSC we identified the position of the nearest known TSS; Figure 8 shows the distribution of the number of TSCs as a function of the relative position of the nearest known mRNA start.

**Figure 9:**
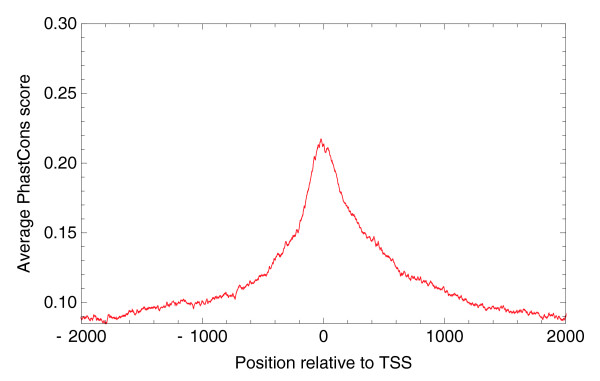
Average PhastCons (conservation) score relative to TSSs of genomic regions upstream and downstream of 'novel' human TSCs that are more than 5 kb away from the start of any known transcript.

By far the most common situation is that there is a known mRNA start within a few base-pairs of the TSC. We also observe a reasonable fraction of cases where a known mRNA start is somewhere between 10 and 100 bp either upstream or downstream of the TSC. Known TSSs more than 100 bp from a TSC are relatively rare and the frequency drops further with distance, with only a few cases of known mRNA starts 1,000 bp away from a TSC. For 37.7% of all TSCs there is no known mRNA start within 1,000 bp of the TSC, and for 27% there is no known mRNA start within 5 kb. We consider these latter 27% of TSCs novel TSCs. To verify that the observed conservation around TSSs shown in Figure 7 is not restricted to TSSs near known mRNA starts, we also constructed a profile of average PhastCons scores around these novel TSCs (Figure 9).

We observe a similar peak to that for all TSCs, although its height is a bit lower and the peak appears a bit more symmetrical, showing only marginally more conservation downstream than upstream of TSSs. Although we can only speculate, one possible explanation for the more symmetrical conservation profile of novel TSCs is that this class of TSCs might contain transcriptional enhancers that show some transcription activity themselves. In Additional data file 1 we present analogous figures for the mouse promoterome.

#### Hierarchical structure of the promoterome

Table 1 shows the total numbers of CAGE tags, TSCs, TSRs, and TSSs within TSCs that we found for the human and mouse CAGE data-sets.

**Table 1:** Global statistics of the human and mouse 'promoteromes' that we constructed from the human and mouse CAGE data

Statistic	Human	Mouse
Number of samples	56	66
Number of mapped CAGE tags	25,469,648	8,104,796
Number of TSSs	6,395,686	1,515,273
Number of TSSs in TSCs	860,823	608,474
Number of TSCs	74,273	77,286
Number of TSRs	43,164	50,915

Shown are the number of different samples, the total number of CAGE tags that were mapped to the genome, the total number of different TSSs that were observed at least once, the number of TSSs in TSCs, the number of TSCs, and the number of TSRs.

The 56 human CAGE samples identify about 74,000 TSCs and the 66 mouse samples identify about 77,000 TSCs. Within these TSCs there are about 861,000 and 608,000 individual TSSs, respectively, corresponding to about 12 TSSs per TSC in human and about 8 TSSs per TSC in mouse. Note that, while large, this number of TSSs is still much lower than the total numbers of unique TSSs that were observed. This again underscores the fact that the large majority of TSSs are expressed at very low levels.

**Figure 10:**
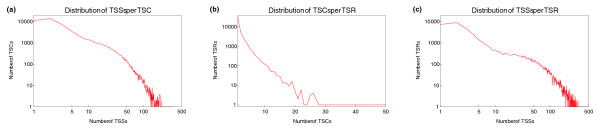
Hierarchical structure of the human promoterome. **(a) **Distribution of the number of TSSs per co-expressed TSC. **(b) **Distribution of the number of TSCs per TSR. **(c) **Distribution of the number of TSSs per TSR. The vertical axis is shown on a logarithmic scale in all panels. The horizontal axis is shown on a logarithmic scale in (a, c).

Next we investigated the hierarchical structure of the human promoterome (similar results were obtained in mouse (see Additional data file 1). Figure 10 shows the distributions of the number of TSSs per TSC, the number of TSSs per TSR, and the number of TSCs per TSR.

Figure 10b shows that the number of TSCs per TSR is essentially exponentially distributed. That is, it is most common to find only a single TSC per TSR, TSRs with a handful of TSCs are not uncommon, and TSRs with more than ten TSCs are very rare. The number of TSSs per TSC is more widely distributed (Figure 10a). It is most common to find one or two TSSs in a TSC, and the distribution drops quickly with TSS number. However, there is a significant tail of TSCs with between 10 and 50 or so TSSs. The observation that the distribution of the number of TSSs per TSC has two regimes is even clearer from Figure 10c, which shows the distribution of the number of TSSs per TSR. Here again we see that it is most common to find one or two TSSs per TSR, and that TSRs with between five and ten TSSs are relatively rare. There is, however, a fairly wide shoulder in the distribution corresponding to TSRs that have between 10 and 50 TSSs. These distributions suggest that there are two types of promoters: 'specific' promoters with at most a handful of TSSs in them, and more 'fuzzy' promoters with more than ten TSSs.

**Figure 11:**
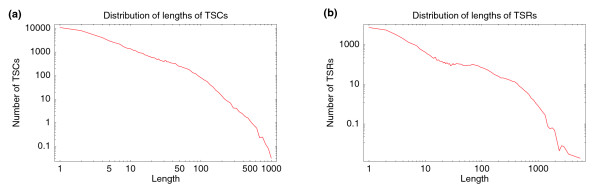
Length (base pairs along the genome) distribution of **(a)** TSCs and **(b)** TSRs. Both axes are shown on logarithmic scales in both panels.

This observation is further supported by the distribution of the lengths of TSCs and TSRs (Figure 11). In particular, the distribution of the length of TSRs (Figure 11b) also shows a clear shoulder involving lengths between 25 and 250 bp or so.

#### Comparison with simple single-linkage clustering

**Figure 12:**
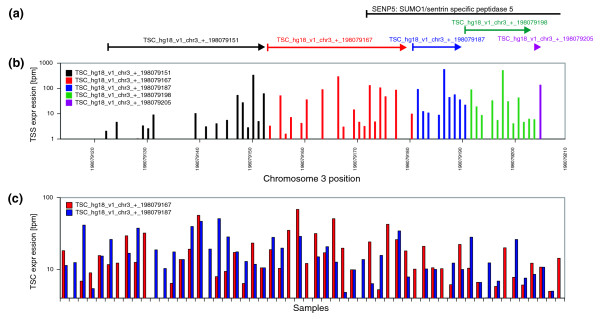
Nearby TSCs with significantly differing expression profiles. **(a) **A 90-bp region on chromosome 3 containing 5 TSCs (colored segments) and the start of the annotated locus of the *SENP5 *gene (black segment). **(b) **Positions of the individual TSSs in the TSC and their total expression, colored according to the TSC to which each TSS belongs. **(c) **Expression across the 56 CAGE samples for the red and blue colored TSCs.

In Additional data file 1 we compare the promoteromes obtained with our clustering procedure with those that were obtained with the simple single-linkage clustering procedures used in FANTOM3. The key difference between our clustering and the single-linkage clustering employed in FANTOM3 is that, in our procedure, neighboring TSSs with significantly different expression profiles are not clustered. Although TSSs within a few base-pairs of each other on the genome often show correlated expression profiles, it is also quite common to find nearby TSSs with significantly differing expression profiles. Figure 12 shows two examples of regions that contain multiple TSSs close to each other on the genome, where some TSSs clearly correlate in expression whereas others do not.

Within a region less than 90 bp wide our clustering identifies 5 different TSCs that each (except for the furthest downstream TSC) contain multiple TSSs with similar expression profiles. Any clustering algorithm that ignores expression profiles across samples would likely cluster all these TSSs into one large TSC. However, as shown in Figure 12c for the red and blue colored TSCs, their expression profiles across samples are not correlated at all. A scatter plot of the expression in TPM of the red and blue colored TSCs is shown in Figure S8 in Additional data file 1, and an additional example analogous to Figure 12 is also shown (Figure S9).

**Figure 13:**
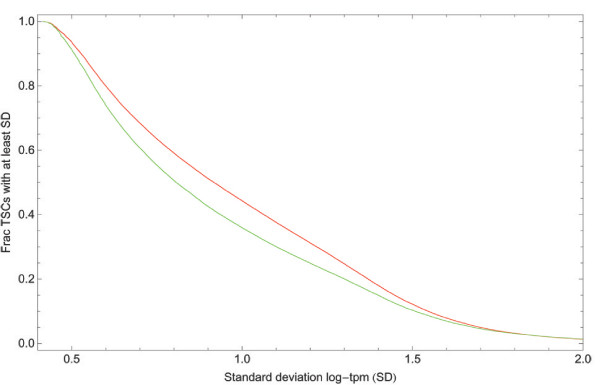
Reverse cumulative distributions of the standard deviation in expression across the 56 CAGE samples for the TSCs obtained with our clustering procedure (red) and the FANTOM3 single-linkage clustering procedure (green).

Since clustering procedures that ignore expression profiles, such as the single-linkage clustering employed in FANTOM3, cluster nearby TSSs with quite dissimilar expression profiles, one would expect that this clustering would tend to 'average out' expression differences across samples. To test this, we calculated for each TSC the standard deviation in expression (log-TPM) for both our TSCs and those obtained with the FANTOM3 clustering. Figure 13 shows the reverse cumulative distributions of the standard deviations for the two sets of TSCs. The figure shows that there is a substantial decrease in the expression variation of the TSCs obtained with the FANTOM3 clustering compared to the TSCs obtained with our clustering. This illustrates that, as expected, clustering without regard for the expression profiles of neighboring TSSs leads to averaging out of expression variations. As a consequence, for TSCs obtained with our clustering procedure one is able to detect significant variations in gene expression, and, thus, potential important regulatory effects that are undetectable when one uses a clustering procedure that ignores expression profiles.

### High and low CpG promoters

**Figure 14:**
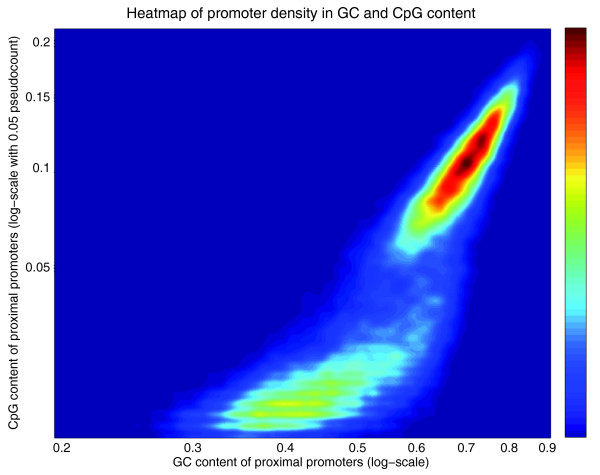
Two-dimensional histogram (shown as a heatmap) of the CG base content (horizontal axis) and CpG dinucleotide content (vertical axis) of all human TSRs. Both axes are shown on logarithmic scales.

Our promoterome statistics above suggest that there are two classes of promoters. That there are two types of promoters in mammals was already suggested in previous CAGE analyses [8], where the wide and fuzzy promoters were suggested to be associated with CpG islands, whereas promoters with a TATA-box tended to be narrow. To investigate this, we calculated the CG and CpG content of all human promoters. That is, for each TSR we determined the fraction of all nucleotides that are either C or G (CG content), and the fraction of all dinucleotides that are CpG (CpG content). Figure 14 shows the two-dimensional histogram of CG and CpG content of all human TSRs.

Figure 14 clearly shows that there are two classes of TSRs with respect to CG and CpG content. Although it has been demonstrated previously that CpG content of promoters shows a bimodal distribution [25], the simultaneous analysis of both CG and CpG content allows for a more efficient separation of the two classes, and demonstrates more clearly that there are really only two classes of promoters. We devised a Bayesian procedure to classify each TSR as high-CpG or low-CpG (Materials and methods) that allows us to unambiguously classify the promoters based on their CG and CpG content. In particular, for more than 91% of the promoters the posterior probability of the high-CpG class was either > 0.95 or < 0.05.

To study the association between promoter class and its length distribution, we selected all TSRs that with posterior probability 0.95 or higher belong to the high-CpG class, and all TSRs that with probability 0.95 or higher belong to the low CpG class, and separately calculated the length distributions of the two classes of TSRs.

**Figure 15:**
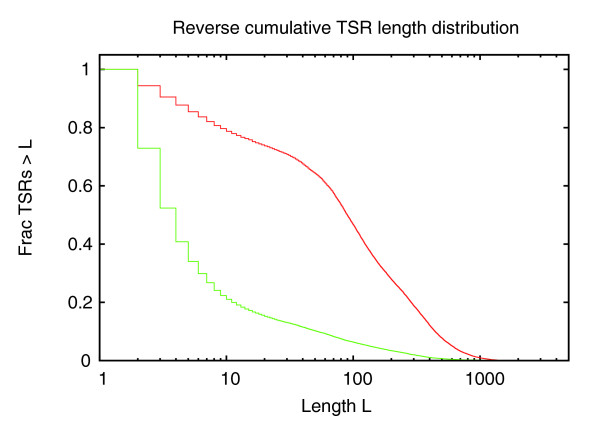
Reverse cumulative distribution of the lengths (base-pairs along the genome) of TSRs for high-CpG (red curve) and low-CpG (green curve) promoters. The horizontal axis is shown on a logarithmic scale.

Figure 15 shows that the length distributions of high-CpG and low-CpG TSRs are dramatically different, supporting observations made with previous CAGE data [8]. For example, for the high-CpG TSRs only 22% have a width of 10 bp or less. In contrast, for the low-CpG TSRs approximately 80% of the TSRs have a width of 10 bp or less. In summary, our analysis supports that there are two promoter classes in human: one class associated with low CpG content, low CG content, and narrow TSRs, and one class associated with high CpG content, high CG content, and wide promoters. Similar results were obtained for mouse TSRs (data not shown).

Finally, we compared the promoter classification of known and novel TSRs. Of the 43,164 TSRs, 37.7% are novel - that is, there is no known transcript whose start is within 5 kb of the TSR. For both known and novel TSRs the classification into high-CpG and low-CpG is ambiguous for about 8% of the TSRs. However, whereas for known TSRs 56% are associated with the high-CpG class, for novel TSRs 76% are associated with the low-CpG class. This is not surprising given that high-CpG promoters tend to be higher and more widely expressed than low-CpG promoters - that is, they are much less likely to not have been observed previously.

## Conclusions

It is widely accepted that gene expression is regulated to a large extent by the rate of transcription initiation. Currently, regulation of gene expression is studied mostly with oligonucleotide micro-array chips. However, most genes initiate transcription from multiple promoters, and while different promoters may be regulated differently, the micro-array will typically only measure the sum of the isoforms transcribed from the different promoters. In order to study gene regulation, it is, therefore, highly beneficial to monitor the expression from individual TSSs genome-wide and deepCAGE technology now allows us to do precisely that. The related RNA-seq technology similarly provides significant benefits over micro-arrays. We therefore expect that, as the cost of deep sequencing continues to come down, deep sequencing technologies will gradually replace micro-arrays for gene expression studies.

Application of deep sequencing technologies for quantifying gene expression is still in its infancy and, not surprisingly, there are a number of technical issues that complicate interpretation of the data. For example, different platforms exhibit different sequencing errors at different rates and, currently, these inherent biases are only partially understood. Similarly, it is also clear that the processing of the input samples to prepare the final libraries that are sequenced introduces biases that are currently poorly understood and it is likely that many technical improvements will be made over the coming years to reduce these biases.

Apart from the measurement technology as such, an important factor in the quality of the final results is the way in which the raw data are analyzed. The development of analysis methods for micro-array data is very illustrative in this respect. Several years of in-depth study passed before a consensus started to form in the community regarding the appropriate normalization, background subtraction, correction for sequence biases, and noise model. We expect that gene expression analysis using deep sequencing data will undergo similar development in the coming years. Here we have presented an initial set of procedures for analyzing deep sequencing expression data, with specific application to deepCAGE data.

Our available data suggest that, across all tissues and conditions, the expression distribution of individual TSSs is a universal power-law. Interestingly, this implies that there is no natural expression scale that distinguishes the large number of TSSs that are expressed at very low rates - so-called background transcription - from the highly regulated expression of the TSSs of highly expressed genes. That is, background transcription and the TSSs of the most highly expressed genes are just the extrema of a scale-free distribution. As we have shown, by assuming that a common universal power-law applies to all samples, we can normalize the expression data from different deep sequencing data-sets. The fact that expression profiles from SAGE and from RNA-seq using the Solexa platform also show power-law distributions strongly suggests that this normalization scheme is applicable to deep sequencing expression data in general. It should be noted that although all observed distributions are power-laws, there is no *a priori *reason that mammalian cells should have a common power-law expression distribution across all tissues and conditions. It is conceivable that, as more extensive data become available in the future, we may find significant differences between the expression distributions in different tissues.

The noise in the expression measured across different deepCAGE samples can be accurately modeled by a convolution of multiplicative noise and Poisson sampling and we derived a practical analytical approximation to the resulting noise distribution. Using replicate data-sets, we inferred the size of the multiplicative noise for different samples and found it to vary in a small range. In addition, analysis of Solexa RNA-seq data from *Drosophila *showed multiplicative noise of similar size. However, we expect that it is a simplification to assume that the size of the multiplicative noise is identical in all experiments, and in the future we will want to apply a more refined analysis that takes into account the differences in the size of the multiplicative noise for different samples. To this end it will be important to design experiments such that at least one replicate is available to estimate the size of the multiplicative noise associated with a given experimental procedure.

The noise model allows us to rigorously assess the statistical significance of measured expression differences across different samples. In particular, we developed a Bayesian procedure that calculates the probability that two TSSs have identical expression profiles. Interestingly, we found that TSSs that are less than 10 bp apart on the genome are much more likely to be co-expressed than more distal neighboring TSSs. Using these results, we clustered sets of nearby co-expressed TSSs into TSCs that we propose are each regulated by a common 'promoter'. Of course, our ability to detect significant expression differences is limited by the number of available samples and we expect that, as the number of available deepCAGE samples increases, the number of TSCs will increase as well.

Comparative genomic analysis shows a strong peak in sequence conservation restricted to a few hundred base-pairs around TSSs. This suggests that the proximal promoter associated with each TSC extends a few hundred base-pairs around the TSSs in the TSC. Besides clustering nearby co-expressed TSSs into TSCs, we also clustered TSCs whose proximal promoters overlap into TSRs. Comparing the sequence composition and widths of TSRs, we find that there are two classes of promoters in the human and mouse genomes. The first class corresponds to TSRs that are narrow, almost always less than 10 bp wide, and that have low CG content as well as low CpG content. The second class corresponds to TSRs that are wide, that is, anywhere from 25 to 250 bp wide, and that are associated with CpG islands, that is, having both a high CG content as well as a high CpG content. It seems plausible that different mechanisms may be involved in the regulation of these two classes of promoters.

## Materials and methods

### CAGE and RNA-seq expression data

All the samples used in this study were provided by the RIKEN Genomic Sciences Center as well as its successor, the Omics Science Center, and come from the FANTOM3, the FANTOM4, and several smaller projects. Each human sample has at least 100,000 mapped tags, and each mouse sample at least 50,000. The lists of all 56 human and 66 mouse samples, with tissue/cell line name, treatment and accession numbers are included in Additional data files 2 and 3. Whenever assigned, accession numbers of the DNA Data Bank of Japan are listed. Raw CAGE data of the FANTOM4 project are available at [9].

The CAGE protocol that was used has been described in [26]. The 143 C6 mouse hippocampus and h93, i02, i03 human THP-1 libraries were produced using a more recent protocol adapted to the 454 Life Sciences sequencer (Roche) as described in the Methods section of [27]. The lengths of the CAGE tags were 20 to 21 bp in all cases.

For the RNA-seq data total RNA was isolated from *Drosophila *Kc cells using Trizol reagent. Purification of mRNA and the generation of the cDNA library were performed following the Illumina protocol for mRNA sequencing. Primary sequencing data analysis was done following the Illumina Genome Analyzer software pipeline. ELAND (part of the Illumina suite) was used for the alignment of short reads to the *Drosophila *genome (release 5).

### Normalization by fitting to a reference distribution

For each CAGE sample we fit the reverse-cumulative distribution *n*(*t*) of the number of TSSs with at least *t *tags to a power-law. To robustly fit these power-laws across different samples with different total numbers of tags, we remove the data from the first and last order of magnitude along the vertical axis and apply simple linear regression to the remaining data. As a result, for each sample *s *there will be a fitted exponent *α *(*s*) and a fitted offset *n*_0 _(*s*).

For a reference distribution of the form *n*_*r *_(*r*_0 _*t*^-*α*^) the total number of tags is given by:

(16)

where *ζ *(*x*) is the Riemann-zeta function. That is, the total number of tags is determined by both *r*_0 _and *α *. For the reference distribution we chose *α *= 1.25 and  = 10^6^. Setting *α *= 1.25 in Equation 16 and solving for *r*_0 _we find:

(17)

To map tag-counts from different samples to this common reference, we transform the tag-count *t *in each sample into a tag-count *t' *according to:

(18)

such that the distribution *n*(*t'*) for this sample will match the reference distribution, that is, *n*(*t'*) = *n*_*r *_(*t'*). If the observed distribution has tag-count distribution:

(19)

then in terms of *t' *this becomes:

(20)

Demanding that *n*(*t'*) = *n*_*r *_(*t'*) gives:

(21)

This equation is satisfied when *α*/*β *= 1.25, that is:

(22)

Using this and solving for *λ *we find:

(23)

### Noise model

We model the noise as a convolution of multiplicative Gaussian noise and Poisson sampling noise. Assume that tags from a given TSS position correspond to a fraction *f *of the tags in the input pool. Let *x *= log(*f*) and let *y *be the log-frequency of the tag in the final prepared sample that will be sequenced, that is, for CAGE after cap-trapping, linking, PCR-amplification, and concatenation. We assume that all these steps introduce a Gaussian noise with variance *σ*^2 ^so that the probability *P*(*y*|*x*,*σ*) is given by:

(24)

We assume that the only additional noise introduced by the sequencing is simply Poisson sampling noise. That is, the probability to obtain *n *tags for this position, given *y *and given that we sequence *N *tags in total is given by:

(25)

Combining these two distributions, we find that the probability to obtain *n *tags given that the log-frequency in the input pool was *x *is given by:

(26)

This integral can unfortunately not be solved analytically. However, if the log-frequency *x *is high enough such that the expected number of tags ⟨*n*⟩ = *Ne*^*x *^is substantially bigger than 1, then the Poisson distribution over *y *takes on a roughly Gaussian form over the area where (*y *- *x*)^2 ^is small enough to contribute substantially to the integral. We thus decided to approximate the Poisson by a Gaussian, that is, we use:

(27)

Then the integral over *y *can be performed analytically. Since the integrand is already close to zero at *y *= 0 (no individual TSS accounts for the entire sample), we can extend the region of integration to *y *= ∞ without loss of accuracy. We then obtain:

(28)

where the variance is given by:

(29)

In summary, the expected tag-count is such that the expected log-frequency log(*n*/*N*) matches the input log-frequency *x*, and has a noise variation of the size *σ*^2 ^plus one over the tag-count *n*.

Although this approximation is strictly only good for large *n*, we find that, in practice, it is already quite good from *n *= 3 or so onwards and we decided to use this approximation for all tag-counts *n*. However, it is clear that for *n *= 0 the approximation cannot be used. For the case *n *= 0 we thus have to make an alternative approximation. The probability *P*(0|*σ*,*x*) is given by the integral:

(30)

We can again extend the integration range to *y *= ∞ without appreciable error. In addition, we introduce a change of variables to:

(31)

and we introduce the variable *m*, which represents the expected number of tags, that is:

(32)

With these definitions the integral becomes:

(33)

The Gaussian second term in the exponent ensures that the main contribution to the integral comes from the region around *z *= 0. We therefore expand *e*^*σz *^to second order, that is:

(34)

The integral then becomes a Gaussian integral and we obtain the result:

(35)

For small *σ *this is in fact very close to:

(36)

Both Equations 35 and 36 are reasonable approximations to the probability of obtaining zero tags given an original log-frequency *x*.

### Estimating the multiplicative noise component from the replicate

Assume a particular TSS position was sequenced *n *times in the first replicate sample and *m *times in the second replicate sample. Assume also that both *n *and *m *are larger than zero. A little calculation shows that the probability *P*(*n*, *m*|*σ*) is given by:

(37)

Note that we have not yet specified if by *n *and *m *we mean the raw tag-counts or the normalized version. For the comparison of expression levels - that is, the difference log(*n*/*N*) - log(*m*/*M*) - it is clear we want to use the normalized values *n' *and *m' *. However, since the normalized values assume a total of 1 million tags, the normalized values cannot be used in the expression for the variance. Therefore, we use the raw tag-counts *n *and *m *in the expression for the variance. That is, the probability takes the form:

(38)

We estimate the variance *σ*^2 ^by maximizing the probability of the data over all positions for which both *n *and *m *are larger than zero. Writing:

(39)

the log-probability *L *of the data can be written as:

(40)

where the sum is over all TSS positions *i*. We can now find the maximum of *L *with respect to *σ*^2^. Doing this on the replicate CAGE data set we find:

(41)

### Estimating the multiplicative noise component by comparing zero and one hour expression in the THP-1 cell PMA time course

Using the assumption that few TSSs change their expression within 1 hour of treatment with PMA, we can also estimate *σ*^2 ^by comparing expression across TSSs in the CAGE samples of THP-1 cells before and after 1 hour of PMA treatment. We assume that a large fraction of the TSS positions should be expressed equally in the two experiments but allow for a small fraction of TSS positions to be expressed differently across the two time points.

Let Δ denote the size of the range in log-expression - that is, the difference between highest and lowest log tag-count - which is about 20,000 in our experiments. We assume a uniform prior distribution *P*(*x*) = 1/Δ over log-frequency *x*. Assume a TSS position has expression *m *at zero hours and *n *at 1 hour. The probability of this expression given that both are expressed the same is *P*(*n, m*|*σ*) that we calculated above (Equation 13). In contrast, if the expression is different between the two time points, then the probability is just the prior 1/Δ. Let *π *denote the (unknown) fraction of all positions that is expressed differently between the two time points. Under these assumptions the likelihood of the data is:

(42)

We now maximize this likelihood with respect to both *π *and *σ*^2^. Doing this on zero and one time points of the three replicates gives us estimated *σ*^2 ^values of 0.048, 0.116, and 0.058. Note that two of these are less than the *σ*^2 ^values inferred from the replicate.

### Likelihood of the expression profile of a single transcription start cluster

We want to calculate the likelihoods of two neighboring TSCs under the assumption that they have fixed relative expression, and assuming the two profiles are independent. As discussed above, the probability of the observed tag-count *n *is, to a good approximation, Gaussian in the log-expression log(*n*) with a variance (*σ*^2 ^+ 1/*n*), where *σ*^2 ^is the variance due to the replicate noise and 1/*n *is the variance due to the Poisson sampling. However, this Gaussian form breaks down when *n *= 0 and this makes analytic derivations impossible when data-points with zero counts are included. To circumvent this, we make two approximations when considering the expression profiles of neighboring TSCs. First, we discard all samples *s *in which both TSSs have zero tag-count *n*_*s *_= 0, that is, we assume, in effect, that samples for which both TSCs have count zero are equally likely under both models. In addition, for samples *s *where one of the two TSCs has a zero count we replace the count zero with a pseudo-count of one-half of a tag (being intermediate between no tags at all and one tag).

We focus first on the probability of the expression profile of a single TSC (considering only the samples in which at least one of the TSCs has non-zero tag count). Let *s *denote a sample, *t*_*s *_the normalized TPM of the TSC in the sample, and *n*_*s *_the un-normalized CAGE tag count in the sample. The log-expression values are given by:

(43)

where the Kronecker delta function is 1 if and only if the tag-count *n*_*s *_is zero and *N*_*s *_is the total number of tags in sample *s *(over all TSSs). We now assume a model of the following form:

(44)

where *μ *is the true average log-expression of this TSC and *δ*_*s *_is the true deviation from this mean in sample *s*. Given our noise model we have:

(45)

where:

(46)

*σ*^2 ^is the variance of the multiplicative noise, and we set *n*_*s *_= 1/2 whenever *n*_*s *_= 0. We need a prior probability distribution for the true expression variation *δ*_*s *_and we will assume this prior to be Gaussian with mean zero, that is, we assume:

(47)

where *α *sets the scale of the variation that TSCs show. As discussed below, we choose *α *so as to maximize the likelihood of all the expression profiles from all TSSs (assuming each TSS is independent).

To obtain the marginal probability of *x*_*s *_given *μ *and *α*, we perform the integral:

(48)

This is a Gaussian integral that can be easily performed and we obtain:

(49)

where:

(50)

Next, to obtain the marginal probability of *x*_*s *_given only *α*, we integrate over the mean log-expression *μ *and to do this we need a prior *P(μ)*. For simplicity we use a uniform prior probability over some fixed range, that is:

(51)

when -Δ_*μ*_/2 ≤ *μ *≤ Δ_*μ*_/2, and zero outside of this range. We then obtain:

(52)

We will assume that Δ*μ *is large compared to the region over which the probability takes on its maximum so that we can let the integral run from minus infinity to infinity without affecting the result. The precise value of Δ*μ *is not important since it will eventually cancel out of the calculation. The result of the integral over *μ *is:

(53)

where *S *is the number of samples (for which at least one of the two neighboring TSCs has non-zero tag-count) and the averages are defined as follows:

(54)

(55)

and

(56)

To estimate *α *we extract, for each TSS *p*, all samples *s *for which the TSS has non-zero tag-count *n*_*s *_and we calculate *P*(*x*|*α*) for each of the expression profiles of these TSSs. The total likelihood of *α *is then simply the product of *P*(*x*|*α*) over all TSSs:

(57)

and we maximize this expression with respect to *α *.

### Likelihood for a consecutive pair of TSCs

The key quantity that we want to calculate is the probability that the expression profiles of two neighboring TSCs are proportional. That is, that the 'true' expression of the one TSC is a constant times the expression of the other TSC. Mathematically, we assume that the means of the log-expressions may be different for the two TSCs, but the deviations *δ*_*s *_are the same. That is, we assume:

(58)

and

(59)

where *x*_*s *_and *y*_*s *_are the log-expression values of the neighboring pair of TSCs. Again, as described above, we restrict ourselves to those samples for which at least one of the neighbors has non-zero expression, and add a pseudo-count of half a tag whenever *n*_*s *_= 0.

For a single sample we have:

(60)

where:

(61)

and *m*_*s *_is the raw tag-count of the TSC with log-expression *y*_*s*_. The integral over *δ*_*s *_is still a Gaussian integral but the algebra is quite a bit more tedious in this case. To simplify the expressions we write:

(62)

and

(63)

Then we can write:

(64)

Next we want to integrate over *μ *and  That is, we want to calculate the integrals:

(65)

where we again use uniform priors:

(66)

Although these integrals are still just Gaussian integrals, the algebra is much more involved. To do the integrals we change variables from *μ *and  to *r *= (*μ *+ )/2 and *q *= *μ *-  (note that the Jacobian determinant of this transformation is 1). We integrate *r *out of the problem first. Furthermore, we introduce notation:

(67)

(68)

(69)

(70)

(71)

and finally

(72)

Using this notation we can write the integral over *r *as:

(73)

where the averages are again defined as:

(74)

(75)

and

(76)

Finally, we integrate over *q*. The result can be written as:

(77)

with

(78)

and all the averages are defined as above. For example, we have:

(79)

and analogously for all the other averages.

### Classifying high- and low-CpG transcription start regions

We first log-transformed the CG and CpG contents of all TSRs. To do this we added a pseudo-count of 0.05 to the fraction of CpG dinucleotides of all TSRs. We fitted (using expectation-maximization) the joint distribution of log-CG and log-CpG contents of all TSRs to a mixture of two two-dimensional Gaussians of the form:

(80)

where the components of  are the logarithms of the fraction of CGs and CpGs, respectively. The fitted solution has:

(81)

The center of the low-CpG Gaussian is given by:

(82)

and the center of the high-CpG Gaussian by:

(83)

The fitted variance of the low-CpG Gaussian is given by:

(84)

and the fitted variance of the high-CpG Gaussian is given by:

(85)

Using the fitted mixture of Gaussians we can calculate, for each TSR at position , the posterior probability that it belongs to the low-CpG class as:

(86)

where *G*_*AT *_() and *G*_*CG *_() are the fitted low-CpG and high-CpG Gaussians, respectively.

### Data availability

The raw data from the FANTOM4 project is available from the FANTOM4 website [28]. The complete human and mouse promoteromes, including the locations of all TSSs, TSCs, TSRs, and their raw and normalized expression profiles across all CAGE samples are available for download from the SwissRegulon web page [29].

## Abbreviations

CAGE: cap analysis of gene expression; ChIP-seq: chromatin immunoprecipitation-sequencing; PMA: phorbol myristate acetate; SAGE: serial analysis of gene expression; TPM: (normalized) tags per million; TSC: transcription start cluster; TSR: transcription start region; TSS: transcription start site.

## Authors' contributions

PB and EvN developed the data normalization procedure, the noise model, and the hierarchical clustering procedure, and applied these to the human and mouse CAGE data. PC and JK produced the CAGE data, CD was responsible for the initial CAGE data processing, and YH coordinated the production and initial processing of the CAGE data. CB performed the *Drosophila *Kc cell RNA-seq experiments and WVB provided the genome mappings. All authors read and approved the final manuscript.

## Additional data files

The following additional data are available with the online version of this paper: a collection of supplementary materials containing 13 supplementary figures and one supplementary table with additional results on the *Drosophila *RNA-seq data, CAGE replicate data, comparison with FANTOM3 clustering, and statistics on the mouse promoterome (Additional data file 1); a table listing all 56 human CAGE samples, with tissue/cell line name, treatment and accession numbers (Additional data file 2); a table listing the analogous data for the 66 mouse CAGE samples (Additional data file 3).

## Supplementary Material

Additional data file 1 Additional results include the *Drosophila *RNA-seq data, CAGE replicate data, comparison with FANTOM3 clustering, and statistics on the mouse promoterome. Click here for file

Additional data file 2 Data for the 56 human CAGE samples, with tissue/cell line name, treatment and accession numbers. Click here for file

Additional data file 3 Data for the 66 mouse CAGE samples, with tissue/cell line name, treatment and accession numbers. Click here for file
