# Supplementary material for: Methods for analyzing deep sequencing expression data: constructing the human and mouse promoterome with deepCAGE data
Source: Genome Biol. 2009 Jul 22;10(7):R79. doi: 10.1186/gb-2009-10-7-r79 (PMC2728533; doi:10.1186/gb-2009-10-7-r79)
Supplement: Additional data file 1 — Additional results include the Drosophila RNA-seq data, CAGE replicate data, comparison with FANTOM3 clustering, and statistics on the mouse promoterome. [file gb-2009-10-7-r79-S1.pdf]

Supplementary materials

Methods for analyzing deep sequencing expression data: Constructing the human and mouse promoterome with deepCAGE data

Piotr J. Balwierz [†], Piero Carninci [‡], Carsten Daub [‡], Jun Kawai [‡], Yoshihide Hayashizaki [‡], Werner Van Belle [$], Christian Beisel [$], Erik van Nimwegen [†,*]

† *Biozentrum, University of Basel, and Swiss Institute of Bioinformatics*
Klingelbergstrasse 50/70, 4056-CH, Basel, Switzerland,
‡ *RIKEN Omics Science Center, RIKEN Yokohama Institute*
1-7-22 Suehiro-cho Tsurumi-ku Yokohama, Kanagawa, 230-0045 Japan
$ *Laboratory of Quantitative Genomics, Department of Biosystems Science and Engineering*
Eidgenössische Technische Hochschule Zurich, Mattenstrasse 26, 4058 Basel, Switzerland
* *Corresponding author. Email: erik.vannimwegen@unibas.ch*

Distributions of reads per position for Solexa RNA-seq data

Using Solexa sequencing we obtained two replicate data-sets of RNA-seq data. After mapping the reads to the genome we determined the distribution of the number of reads per position for each replicate. Figure S1 shows the reverse cumulative distributions of reads per position that we obtained for these data sets. The figure illustrates that approximately power-law distributions are observed for RNA-seq data as well. This further supports that the roughly power-law distribution of expression levels across individual TSSs is not an artifact of measurement technology but represents the actual distribution of transcript levels in the cells.

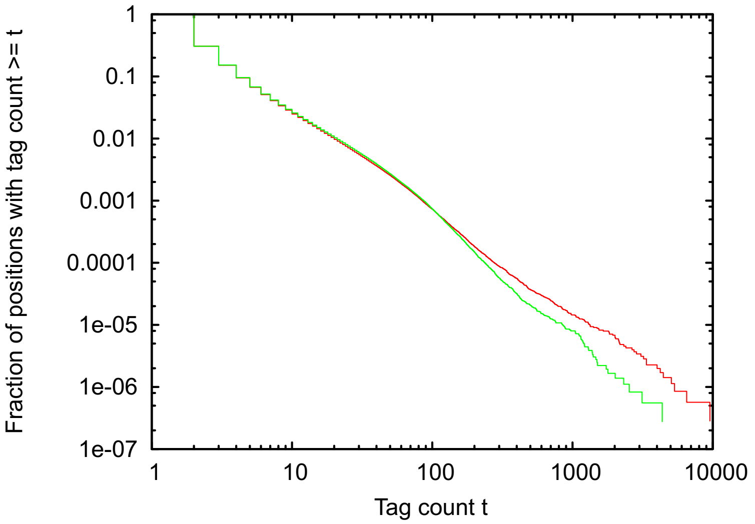

Figure S1: Reverse cumulative distributions for the number of reads per position in two RNA-seq technical replicates of Drosophila Kc cells. Both axes are shown on logarithmic scales.

Replicate scatter for Solexa RNA-seq data

For the same two RNA-seq samples figure S2 shows a scatter-plot of the number of reads per position in the two samples.

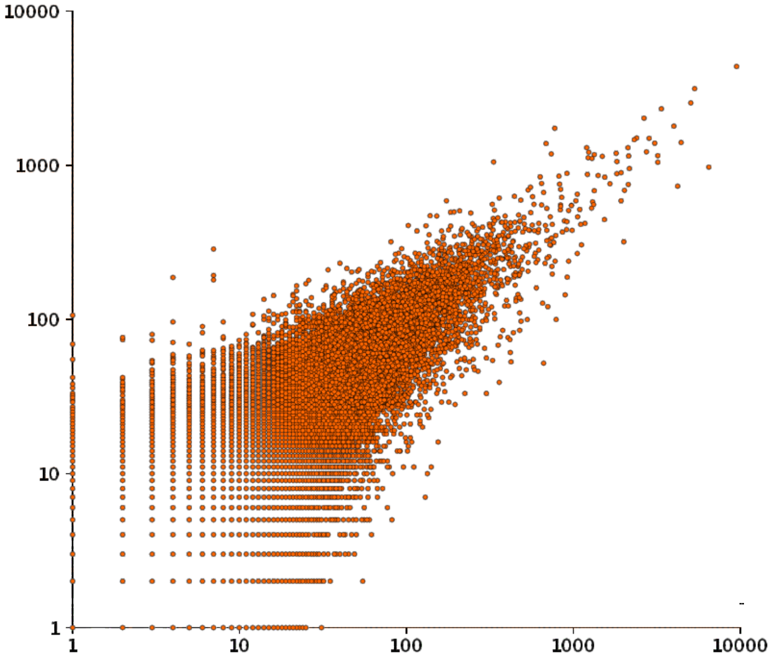

Figure S2: Scatter-plot of numbers of reads in the two RNA-seq replicates of Drosophila Kc cells obtained with Solexa sequencing. Each data point corresponds to a unique position on the chromosome with the number of reads in the first replicate on the horizontal axis and the number of reads in the second replicate on the vertical axis. Both axes are shown on a logarithmic scale. The size of the multiplicative noise σ^2 estimated from this scatter is $\sigma^2 = 0.073$.

Per `exon' replicate scatter for Solexa RNA-seq data

For the same data-set shown in figure S2 we used single-linkage clustering to cluster overlapping reads into `exons'. Figure S3 shows a scatter plot analogous to figure S2 but now for the expression of these `exons' across the two replicates.

CAGE per TSS replicate scatter

Two independent CAGE samples were obtained from a common RNA sample from THP-1 cells after 8 hours of treatment with LPS. Figure S4 shows a scatter-plot of the normalized tags-per-million of each TSS for these two replicate samples.

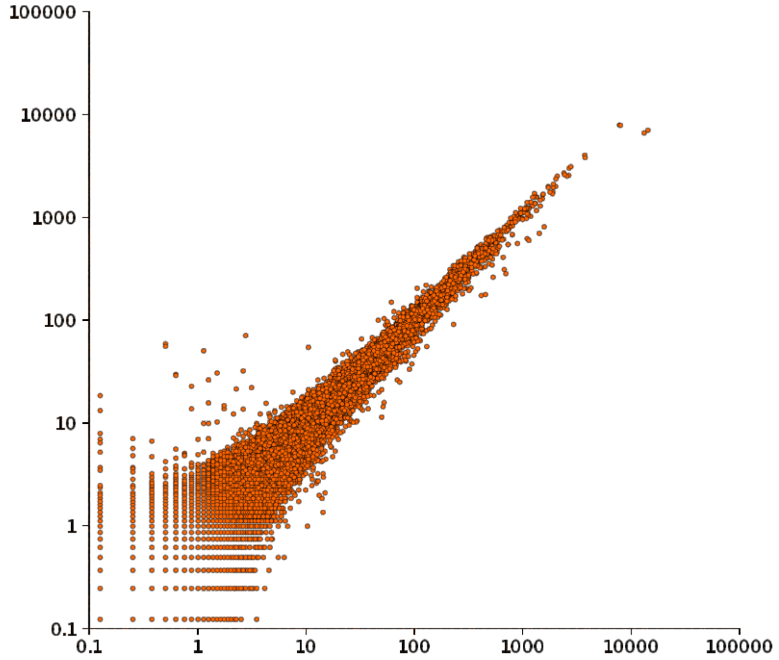

Figure S3: Scatter-plot of reads per million in two RNA-Seq replicates of Drosophila Kc cells. Each data point corresponds to a cluster of overlapping reads on the chromosome, with horizontal and vertical coordinates given by the number of reads per million for each replicate. Both axes are shown on a logarithmic scale. The size of the multiplicative noise σ^2 estimated from this data is $\sigma^2 = 0.02$.

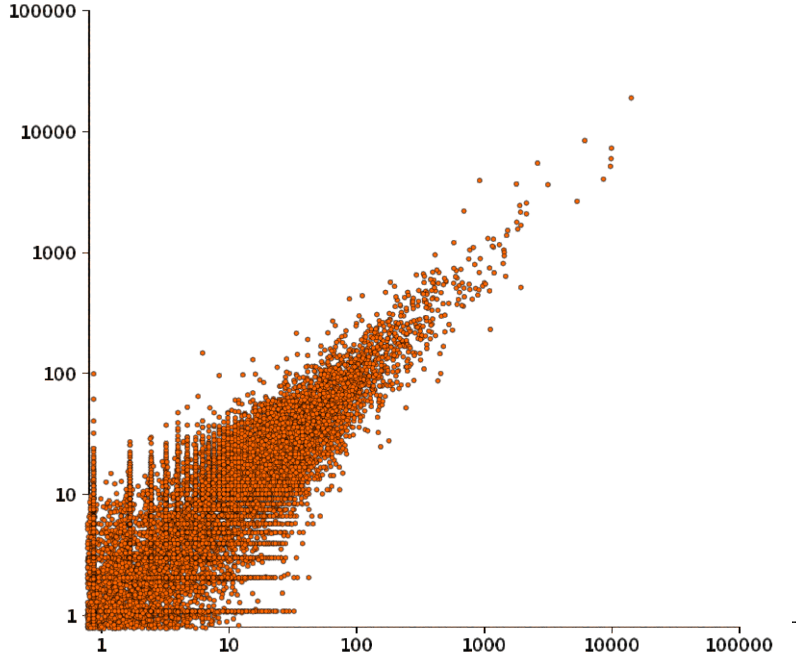

Figure S4: Scatter-plot of CAGE expression for two replicate measurements of THP-1 cells after 8 hours of LPS treatment. Each data point corresponds to a individual TSS. Values on the horizontal and vertical axes correspond to normalized tags per million for each TSS. Both axes are shown on a logarithmic scale. The size of the multiplicative noise estimated σ^2 from this data is $\sigma^2 = 0.085$.

CAGE per gene replicate scatter

For the same two replicate samples shown in figure S4 we summed, for each gene, the expression from all TSSs associated with the gene, to obtain a normalized expression per gene. Figure S5 shows a scatter-plot of the per gene expression of the CAGE replicates.

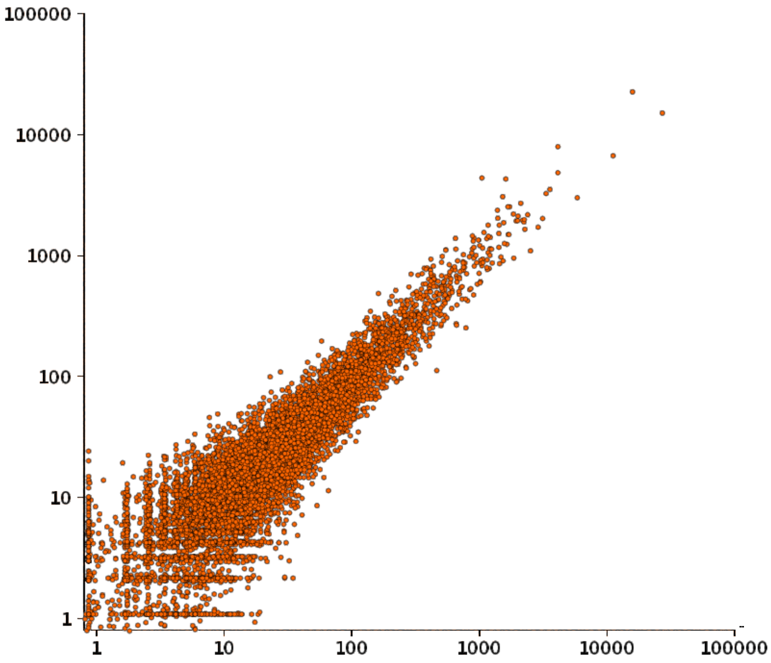

Figure S5: Scatter-plot of the normalized tags per million *per gene* for the same two CAGE replicates as shown in Fig. S4. Each data point corresponds to a gene. Axes are shown on logarithmic scales. The size of the multiplicative noise σ^2 estimated from this data is $\sigma^2 = 0.068$

Comparison with FANTOM3 clustering

For human our data contained a total of $25,469,648$ CAGE tags representing $6,395,686$ unique TSS locations in the human genome. Table S1 compares the number of TSSs in TSCs, the number of TSCs, and the number of TSRs between our clustering of CAGE tags and the simple single-linkage clustering employed in the FANTOM3 paper.

Statistic	Our clustering	FANTOM3 clustering
Number of TSSs in TSCs	860,823	1,043,768
Number of TSCs	74,273	64'908
Number of TSRs	43,164	49'461

Table S1: Comparison of the number of TSSs, TSCs, and TSRs obtained with our clustering and the FANTOM3 clustering (in which CAGE tags that are 21 bp or less apart are clustered through single-linkage clustering).

First of all we see that a significantly larger number of unique TSSs are included in the FANTOM3 clustering. This is a result of the fact that TSSs with expression profiles significantly different from those in the TSC (which may often be low expressed TSSs) are clustered with the TSC in the FANTOM3 clustering, whereas in our clustering these form separate TSCs who are then filtered out owing to their low expression. The total number of TSCs in the FANTOM3 clustering is lower because neighboring TSCs with different expression profiles are all clustered together in the FANTOM3 clustering. Even though the number of TSCs is smaller in the FANTOM3 clustering, the final number of TSRs is a little larger because, owing to the tendency of the FANTOM3 clustering to cluster all nearby TSSs, irrespective of their expression profile, a large number of low expressed TSRs pass the cut-off on minimal expression in the filtering stage.

Figure S6 shows a comparison of the distributions of the number of TSSs per TSC, the number of TSCs per TSR, and the number of TSSs per TSR, for our clustering and for the single-linkage clustering that was employed in FANTOM3.

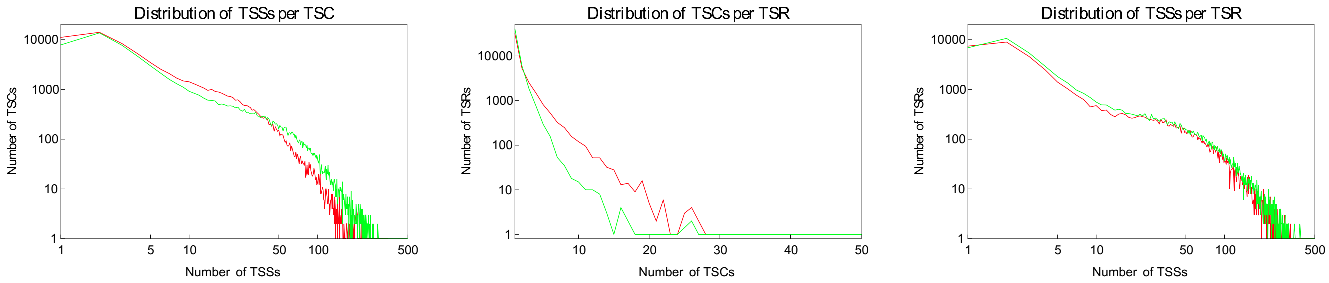

Figure S6: Comparison of the hierarchical structure of the human promoterome for our clustering and the FANTOM3 clustering. Left: Distribution of the number of transcription start sites (TSSs) per co-expressed transcription start cluster (TSC). Middle: Distribution of the number of TSCs per transcription start region (TSR). Right: Distribution of the number of TSSs per TSR. The vertical axis is shown on a logarithmic scale in all panels. The horizontal axis is shown on a logarithmic scale in the left and right panels. The red lines show the distributions obtained using our clustering procedure and the green lines show the distribution obtained using single-linkage clustering employed in FANTOM3

As illustrated by the left and right panels of figure S6, there are in general more TSSs per TSC and more TSSs per TSR for the FANTOM3 clustering. In contrast, there tend to be more TSCs per TSR for our clustering. Both these observations are a result of the fact that in our clustering TSSs with different expression profiles are not clustered together, even if they are near each other, whereas the single-linkage clustering fuses all these TSSs into a single TSC.

Figure S7 shows the distributions of the lengths TSCs and TSRs for both our clustering and the FANTOM3 clustering. Although on the logarithmic scales the length distributions appear quite similar for the two clustering procedures, the TSCs obtained by the FANTOM3 clustering tend to be significantly wider. More strikingly, for the FANTOM3 clustering there is a pronounced shoulder in the distributions at a width of 21 base pairs, which is almost certainly an artifact of the fact that this distance is precisely the cut-off distance on the single-linkage clustering.

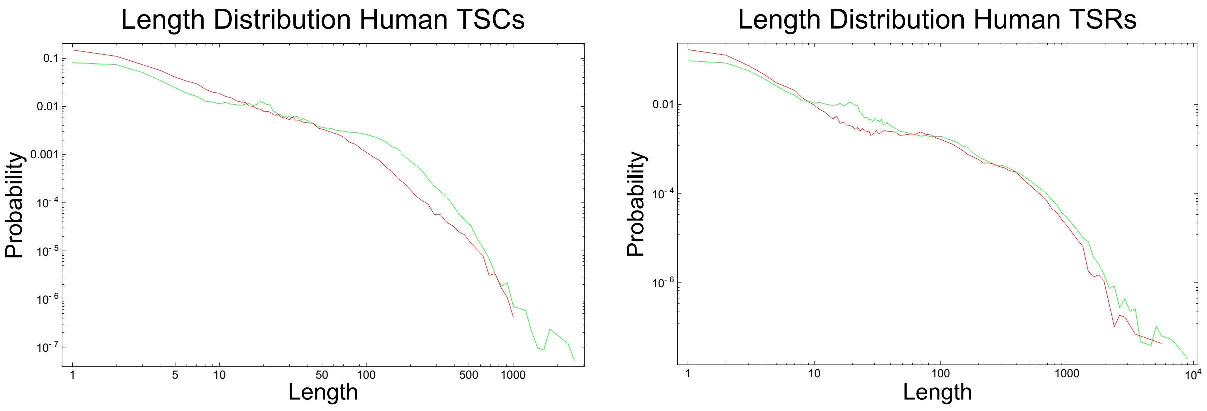

Figure S7: Comparison of the length distributions of TSCs and TSRs for the promoteromes obtained using our clustering and using the FANTOM3 clustering. Left: Length distribution of the TSCs. Right: Length distribution of the TSRs. Both axes are shown on logarithmic scales. The red lines show the distributions obtained using our clustering procedure and the green lines show the distributions obtained using the single-linkage clustering employed in FANTOM3.

Nearby uncorrelated TSSs

In figure 12 of the main article we showed an example of neighboring TSCs that have significantly different expression profiles, which were shown in panel C. To further illustrate that these expression profiles are indeed not correlated figure S8 shows a scatter plot of the expression of the two TSCs across the 56 CAGE samples. The plot confirms that there is no discernible correlation between the expression profiles of the two TSCs, and they are certainly not tightly co-regulated, which supports that these two TSCs are driven by distinct sets of regulatory sites.

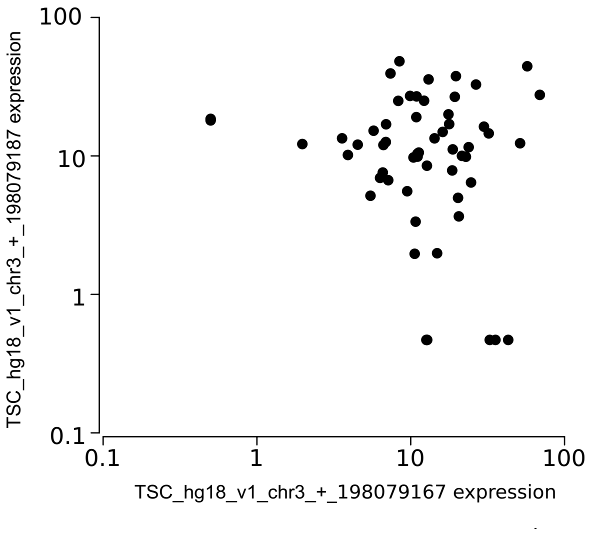

Figure S8: Scatter of the expression levels (in TPM) of two nearby TSCs, located on human chromosome 3. Each dot corresponds to one of the 56 human CAGE samples. Both axes are shown on a logarithmic scale.

In figure S9 we show another example of a set of nearby TSCs with clearly distinct expression profiles. The interesting feature of this example is that there are two broad TSCs, containing a substantial number of TSSs that all show correlated expression, which are interspersed by a *single* TSS that shows a very different expression profile (the red TSS). The structure of this promoter region suggests that, on the one hand, there is a broad region to which the polymerase is recruited by one set of regulatory mechanisms, while on the other hand there is a single TSS within the same region to which the polymerase is recruited by a distinct regulatory mechanism.

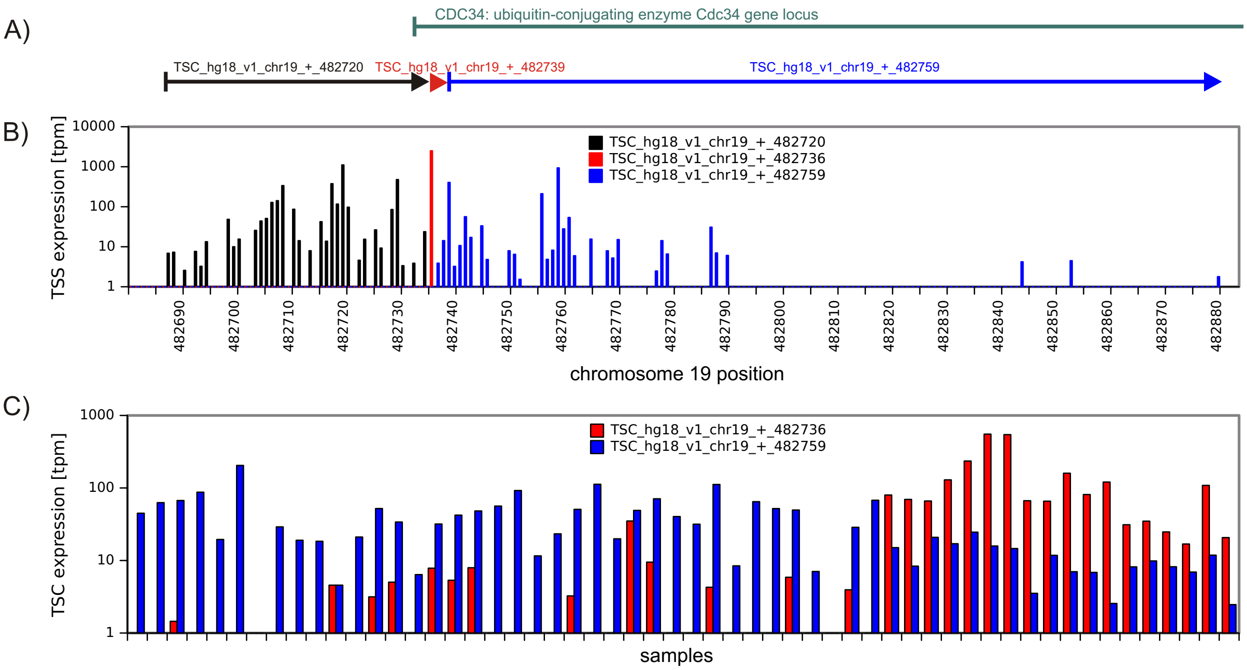

Figure S9: Nearby TSCs with significantly differing expression profiles. (A) An approximately 200 base pair region on chromosome 9 containing 3 TSCs (colored segments) and the start of the annotated locus of the CDC34 gene (black segment). (B) Positions of the individual TSSs in the TSC and their total expression, colored by the TSC to which each TSS belongs. (C) Expression across the 56 CAGE samples for the red and blue TSCs.

Mouse Promoterome Statistics

For the mouse promoterome, as for the human promoterome, we first calculated the distribution of phastCons conservation scores as a function of position relative to the most expressed TSS in each TSC. Figure S10 shows the phastCons conservation profiles that we obtained for both all TSCs (left panel) and the novel TSCs (right panel).

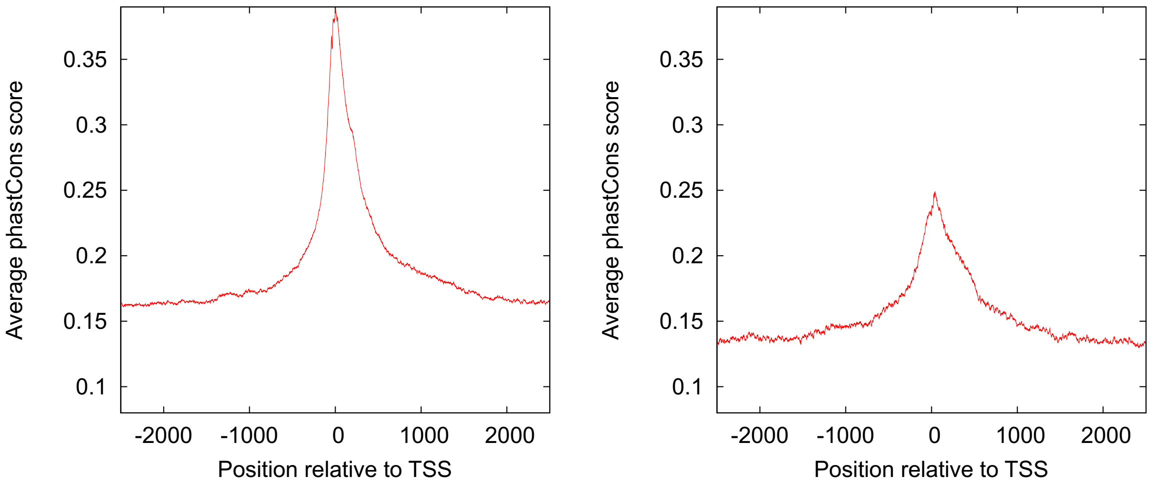

Figure S10: Average phastCons (conservation) score relative to TSS of genomic regions upstream and downstream of all mouse TSCs (left panel) and for all novel mouse TSCs that are more than 5 kilobases away from any known start (right panel).

The conservation profiles for mouse are very similar to the ones that we observed for human. We again see a sharp peak of conservation covering a few hundred base pairs around TSS. The novel promoters show a conservation peak of similar width but with lower height. Interestingly, whereas for human the conservation peak of the novel promoters was close to symmetric, for mouse the novel promoter peak is also clearly asymmetric, although still not as asymmetric as the peak for the known TSSs.

Next we determined the position of the closest start of a known transcript for each mouse TSC. Figure S11 shows the distribution of the relative positions of the closest known starts for all mouse TSCs that have a known start within 1000 base pairs of the TSC.

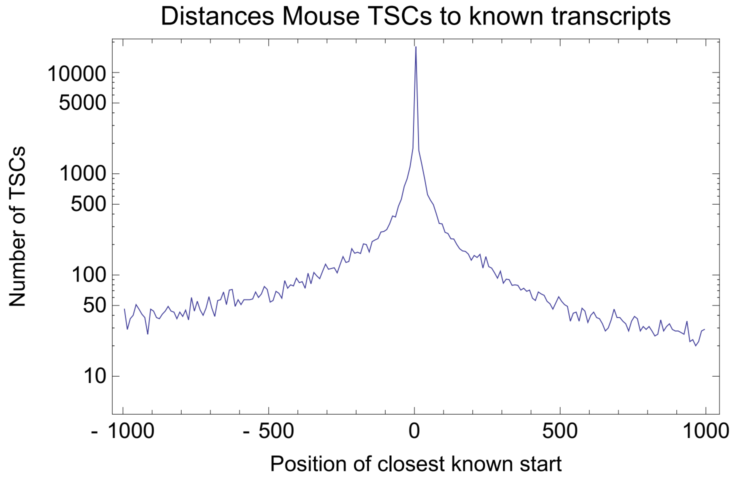

Figure S11: Number of TSCs as a function of their position relative to the nearest known transcript start.

Negative numbers mean the nearest known start is upstream of the TSC. The vertical axis is shown on a logarithmic scale. The figure shows only the $45,603$ TSCs (59%) with a known start within 1000 base pairs.

The distribution in figure S11 is also very similar to what we observed for the human promoterome. The main difference is that whereas for human 62.2% of all TSCs have a known start within 1000 base pairs, for mouse this is only 59%, which is likely due to the larger amount of data available for human.

Figure S12 shows the hierarchical structure of the mouse promoterome that we constructed. In particular, we show the distribution of the number of TSSs per TSC, the number of TSCs per TSR, and the number of TSSs per TSR, as we also showed for the human promoterome in the main article.

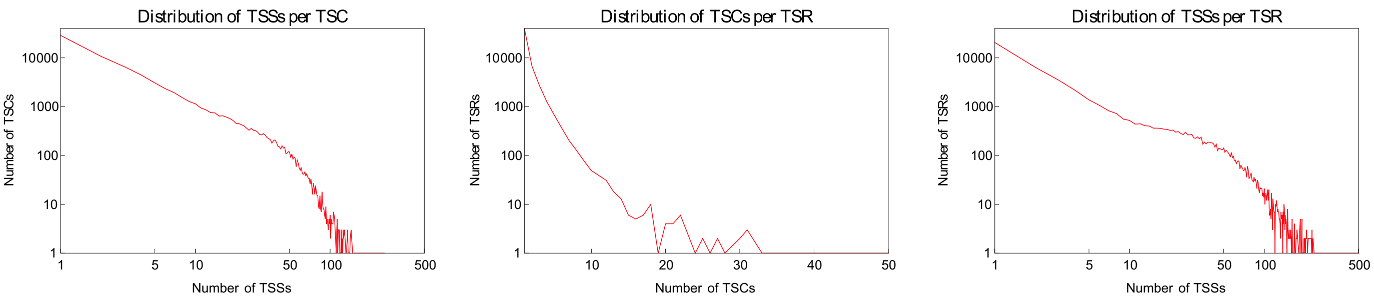

Figure S12: Hierarchical structure of the mouse promoterome. Left: Distribution of the number of transcription start sites (TSSs) per co-expressed transcription start cluster (TSC). Middle: Distribution of the number of TSCs per transcription start region (TSR). Right: Distribution of the number of TSSs per TSR. The vertical axis is shown on a logarithmic scale in all panels. The horizontal axis is shown on a logarithmic scale in the left and right panels.

The distributions in figure S12 are generally very similar to those observed for the human promoterome. The distributions are all a little less wide than for human, which is likely the result of the larger amount of data available for human. Importantly, as in the human data, the distribution of the number of TSSs per TSR also shows the clear `shoulder' corresponding to TSRs with between roughly 10 and 50 TSSs.

Finally, we also calculated the length distributions of mouse TSCs and TSRs, both using our clustering procedure, and using the single-linkage clustering employed in FANTOM3 (figure S13). Here too the distributions are very similar to the results that we obtained for the human data. In particular, we clearly see the shoulder in the distribution of TSR lengths for lengths roughly between 25 and 150 base pairs long. We also again see that the single-linkage clustering leads to wider clusters, and leads to an artificial shoulder at 21 base pairs (i.e. the length of the CAGE tags that was chosen as a distance cut-off).

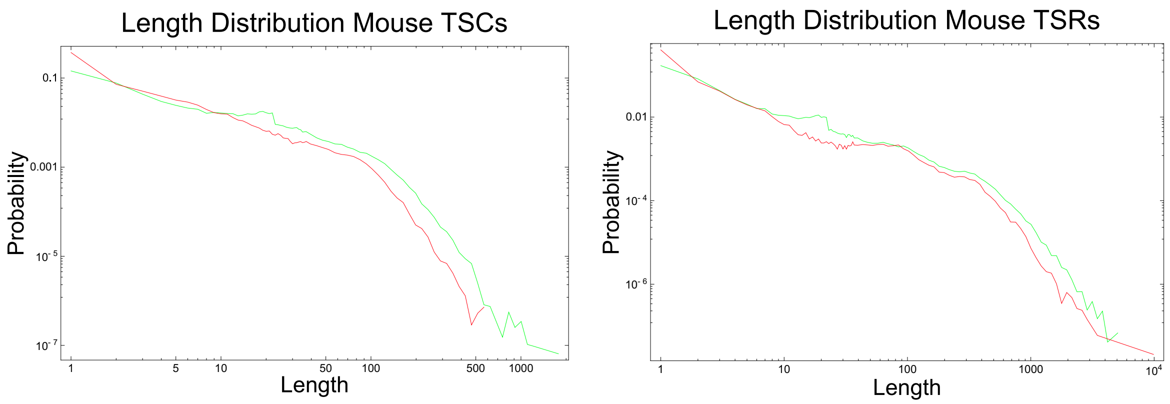

Figure S13: Comparison of the length distributions of TSCs and TSRs for the mouse promoteromes obtained using our clustering and using the FANTOM3 clustering. Left: Length distribution of the TSCs. Right: Length distribution of the TSRs. Both axes are shown on logarithmic scales. The red lines show the distributions obtained using our clustering procedure and the green lines show the distribution obtained using single-linkage clustering employed in FANTOM3.
